# Humanistic Health Management and Cancer: Associations of Psychology, Nutrition, and Exercise with Cancer Progression and Pathogenesis

**DOI:** 10.1002/advs.202400665

**Published:** 2024-03-25

**Authors:** Chenchen Li, Junfeng Zhang, Pengcheng Pan, Junjie Zhang, Xinyi Hou, Yan Wang, Guoping Chen, Pir Muhammad, Rui L. Reis, Lin Ding, Yanli Wang

**Affiliations:** ^1^ International Joint Research Center of Human‐machine Intelligent Collaborative for Tumor Precision Diagnosis and Treatment of Hainan Province & Key Laboratory of Tropical Translational Medicine of Ministry of Education School of Pharmacy & The First Affiliated Hospital Hainan Medical University Haikou 571199 P. R. China; ^2^ Tumor Precision Targeting Research Center & Institute of Nanochemistry and Nanobiology School of Environmental and Chemical Engineering Shanghai University Shanghai 200444 P. R. China; ^3^ 3B's Research Group I3Bs‐Research Institute on Biomaterials Biodegradables and Biomimetics University of Minho Guimarães 4805‐017 Portugal; ^4^ Translational Medicine Collaborative Innovation Center Shenzhen People's Hospital (The First Affiliated Hospital, Southern University of Science and Technology The Second Clinical Medical College of Jinan University) Shenzhen Guangdong 518055 P. R. China; ^5^ Guangdong Engineering Technology Research Center of Stem Cell and Cell Therapy Shenzhen Key Laboratory of Stem Cell Research and Clinical Transformation Shenzhen Immune Cell Therapy Public Service Platform Shenzhen 518020 P. R. China

**Keywords:** cancer, exercise, humanistic health management, nutrition, psychology

## Abstract

The incidence rate of cancer is increasing year by year due to the aging of the population, unhealthy living, and eating habits. At present, surgery and medication are still the main treatments for cancer, without paying attention to the impact of individual differences in health management on cancer. However, increasing evidence suggests that individual psychological status, dietary habits, and exercise frequency are closely related to the risk and prognosis of cancer. The reminder to humanity is that the medical concept of the unified treatment plan is insufficient in cancer treatment, and a personalized treatment plan may become a breakthrough point. On this basis, the concept of “Humanistic Health Management” (HHM) is proposed. This concept is a healthcare plan that focuses on self‐health management, providing an accurate and comprehensive evaluation of individual lifestyle habits, psychology, and health status, and developing personalized and targeted comprehensive cancer prevention and treatment plans. This review will provide a detailed explanation of the relationship between psychological status, dietary, and exercise habits, and the regulatory mechanisms of cancer. Intended to emphasize the importance of HHM concept in cancer prevention and better prognostic efficacy, providing new ideas for the new generation of cancer treatment.

## Graphical abstract

The occurrence of cancer is directly related to personal life habits. Through self‐health management, cancer can be effectively prevented and treated. Therefore, the concept of humanistic health management is put forward, which is a new personalized and comprehensive cancer prevention and control program involving individual psychological conditions, living habits, diet structure, sports characteristics, and other aspects. This review systematically discusses the relationship between psychology, nutrition, exercise, and cancer prevention, occurrence, and development. The aim is to improve people's view of the relationship between daily behavior and cancer from the perspective of overall development, and consciously practice the humanistic health concept.

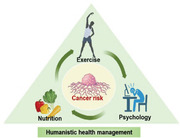

## 1 Introduction

Cancer has always been a major public health problem worldwide. The latest global cancer burden data for 2020 released by the International Agency for Research on Cancer (IARC) indicated that there were 19.29 million new cancer cases and 9.96 million deaths worldwide in 2020. Among them, China had 4.57 million new cancer cases and 3 million deaths, both ranking first in the world.^[^ ^1^ ^]^ According to the national cancer statistics released by the National Cancer Center of China, the incidence and death rate of malignant tumors in China continues to rise, indicating that the country is facing a serious cancer burden.^[^ ^2^ ^]^

The results of the rural‐urban analysis showed that the morbidity in urban areas of China was slightly higher than that in rural areas, while the mortality rate was reversed.^[^ ^2^ ^]^ The slightly higher urban incidence was due to the higher risk of exposure to malignant cancer risk factors such as smoking, chronic infections, dietary habits, and air pollution. However, the relative lack of medical resources in rural areas and the weak awareness of cancer prevention led to the high mortality rate of malignant tumors in rural areas. With the progress of medical technology, compared with 10 years ago, the overall survival rate of malignant tumors in China has increased by ≈10%, but there is still a big gap with developed countries. For example, the 5‐year survival rate of tumors with good prognosis in China, such as breast cancer (82.0%), thyroid cancer (84.3%), and prostate cancer (66.4%), still lags behind that of developed countries such as the United States (90.9%, 98%, and 99.5%).^[^ ^2^ ^]^ The 2023 Cancer Report noted that since 1991, cancer death rates in the United States have continued to decline, with an overall decline of 33% and an estimated 3.8 million cancer deaths averted. This steady progress was due to a decline in smoking, widespread access to early screening for breast, colorectal, and prostate cancers, coupled with the enhancement of therapeutic methods.^[^ ^3^ ^]^

Data originating from the United States underscores the pivotal role of cancer prevention, alongside early diagnostics and therapeutic interventions, in significantly mitigating the incidence and fatality rates associated with cancer.^[^ ^4^ ^]^ Surveys showed that only ≈5%–10% of cancer cases were inherited through abnormal genes, meaning that most cancers are preventable.^[^ ^5^ ^]^ Some of the major risk factors for cancer may include unhealthy eating habits, long‐term acidosis, smoking, alcohol consumption, sleep disturbances, infections, stress, lack of exercise, and exposure to environmental factors such as toxins and radiation.^[^ ^6^ ^]^ From 2004 to 2018, China's obesity rate rose from 3.1% to 8.1%, and modifiable risk factors such as unhealthy lifestyles, obesity, and physical inactivity account for more than 40% of cancer incidence and mortality in China.^[^ ^7^ ^]^ The downward trend in liver cancer incidence may be due to a decrease in the consumption of foods contaminated with aflatoxins.^[^ ^8^ ^]^ The above evidence further indicates that individual lifestyle habits are directly related to the risk of cancer, and the incidence of cancer can be effectively reduced by improving self‐health management. In China, especially, when facing the severe situation of large urban‐rural gap and regional imbalance, improving people's awareness of cancer prevention and advocating self‐health management and regular physical examination can reduce the occurrence of cancer from the source.

In 2008, China's Ministry of Health launched the “Healthy China 2020” strategic study, which first put forward the concept of “One Health”. One Health is a global concept put forward according to the development of The Times, social needs, and changes in the spectrum of diseases. It revolves around the relationship between people's lifestyle and disease and aging, pays attention to various risk factors and misunderstandings that affect health, advocates self‐health management, and is put forward under the guidance of the concept of comprehensive care for the whole process of life. It seeks not only individual physical health but also spiritual, psychological, physiological, social, environmental, moral, and other aspects of complete health. Advocate not only scientific healthy life but also correct health consumption and so on. It covers all types of health‐related information, products, and services, as well as the actions taken by organizations to meet the health needs of society. The proposal of the concept of One Health indicates that China and even the world pay more attention to health. The content of health includes not only physical health but also mental health and spiritual health. It expresses the quality of human life existence and is a “positive lifestyle” involving individuals, the environment, and society. The concept of One Health also plays a guiding role in the prevention and treatment of cancer.

**Figure 1:**
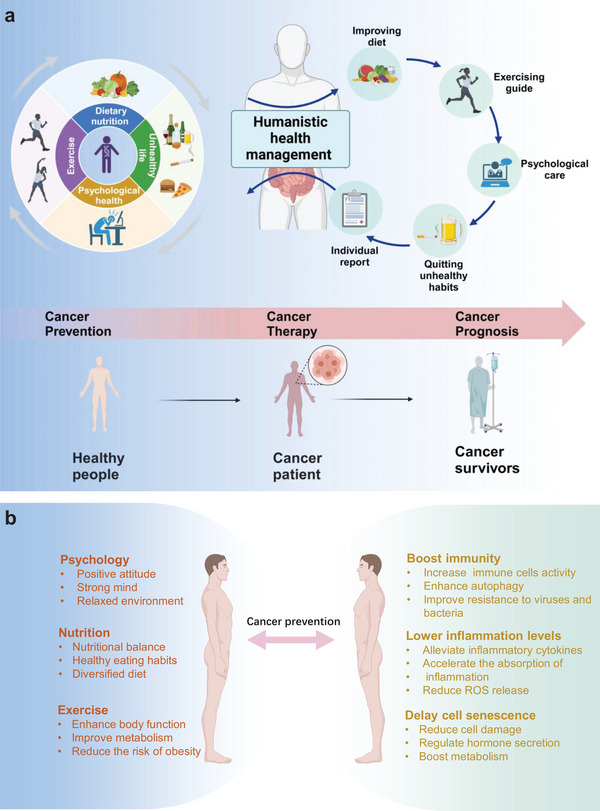
a) The concept of humanistic health management (HHM). Psychology, nutrition, and exercise, as the three components of HHM, interact to form a complex system. The three elements complement each other and work together to improve body functions and anti‐tumor immunity, thereby preventing and treating cancer and improving cancer prognosis. To maintain a healthy lifestyle, it is necessary to consider these three aspects in a comprehensive manner and take appropriate measures to promote a virtuous cycle among them. b) Summary diagram of the relationship between psychology, nutrition, exercise, and cancer prevention.

Under the circumstances, we put forward the concept of “Humanistic Health Management” (HHM). This concept is centered around health management, and based on multiple aspects such as individual psychological status, lifestyle habits, dietary structure, and exercise characteristics, precise and comprehensive evaluations are made to develop personalized and targeted cancer prevention and treatment plans. On the one hand, through health management, to prevent and reduce the risk of cancer. On the other hand, for the treatment of cancer prognosis, the philosophy emphasizes patient‐centered care and personalized medicine (**Figure** **1**a).

Psychology, nutrition, and exercise are three important components of HHM, and they have important connections with the occurrence and development of cancer (Figure 1b).^[^ ^9^ ^]^ For example, a positive mindset could help patients cope with the emotional and psychological impact of a cancer diagnosis and can also improve treatment adherence and overall quality of life.^[^ ^10^ ^]^ On the contrary, anxiety, tension, depression, and other psychological factors seriously affect the prognosis of patients.^[^ ^11^ ^]^ A healthy diet can help support the body's immune system and improve overall health.^[^ ^12^ ^]^ Exercise can improve physical function, reduce fatigue, and enhance anti‐tumor immunity.^[^ ^9^, ^13^ ^]^ Maintaining a good mental attitude, healthy eating habits, and proper exercise habits can effectively prevent cancer and improve cancer prognosis survival.

This review will elaborate on the regulatory mechanisms of psychology, nutrition, and exercise in the occurrence and development of cancer, promote the HHM concept, enhance people's understanding and attention to the relationship between self‐health management and cancer progression, propose a new concept of comprehensive health in China, and integrate individual lifestyles into cancer prevention, diagnosis, treatment, and rehabilitation. It will provide new ideas for reducing the incidence rate of cancer and improving the cure rate of cancer patients.

## 2 The Relationship Between the 3 Main Elements of HHM: Psychology, Nutrition, Exercise and Cancer

According to the World Health Organization (WHO), “Up to 80% of cancers are caused by external factors, such as lifestyle and environmental factors.” Recently, the American Cancer Society released “Cancer Prevention & Early Detection Facts & Figures 2023–2024” to analyze the risk factors for cancer occurrence and death in the United States. According to the report, 1958310 new cases of cancer are expected to occur in the United States in 2023, resulting in 609820 deaths. Of these, 42% of new cancers and 45% of cancer deaths are related to unhealthy lifestyle habits such as smoking, alcohol consumption, physical inactivity, unhealthy diet, overweight or obesity, and long‐term exposure to UV radiation (**Table** **1**).

**Table 1:** The main risk factors for cancer occurrence and death in the United States 2023–2024.

Risk factors	Proportion of deaths	Related cancer types
Tobacco	30%	Lung cancer, oral cancer, pharyngeal cancer, larynx cancer, esophageal cancer, pancreatic cancer, etc
Overweight or obese	Men is 5% women are 11%	Column endometrial cancer, esophageal adenocarcinoma, liver cancer, gastric cardia cancer, kidney cancer, meningioma, multiple myeloma, pancreatic cancer, etc
Physical inactivity	3%	Colon cancer, endometrial cancer, lung cancer, breast cancer, kidney cancer, bladder cancer, esophageal cancer
Unhealthy diets	4%−5%	Colon cancer, stomach cancer, esophageal cancer
Alcohol drinking	5%−6%	Oral cancer, throat cancer, esophageal cancer, liver cancer, rectal cancer, colon cancer, breast cancer, etc

Tobacco use is the leading preventable cause of cancer and death in the United States, contributing to ≈30% of cancer deaths. People who successfully quit smoking can add up to 10 years to their life expectancy. For cancer survivors, quitting smoking is also associated with longer survival and better quality of life outcomes. About 5% and 11% of new cancers in men and women are caused by being overweight or obese. Being overweight or obese increases the risk of 13 types of cancer and death. About 3% of new cancer cases are linked to physical inactivity, and the more sedentary the higher the risk of colon, endometrial, and lung cancers. Sitting has also been linked to an increased risk of cancer death. Increased physical activity may reduce the risk of colon, breast, kidney, endometrial, bladder, esophageal adenocarcinoma, and lung cancers. 4%−5% of new cancers are linked to unhealthy diets. Regular consumption of red meat, processed meats, starchy foods, refined grains, and sugary drinks has been linked to an increased risk of cancer, particularly colon cancer. Those who regularly ate a variety of fruits and vegetables, whole grains, legumes, fish or poultry, and less red and processed meat had a reduced risk. Between 5% and 6% of new cancers are linked to alcohol use. Cancer risk increases with the amount of alcohol consumed, and even small amounts of alcohol are associated with an increased risk of cancer. In addition, if smoking and drinking alcohol are combined, they lead to a greater risk of cancer, far exceeding the risk of cancer caused by smoking or drinking alone.

In addition to the above risks, in recent decades, long‐term stress in life and society, resulting in psychological factors is also associated with the risk of cancer. Through experimental studies, many researchers found that emotional states and their accompanying physiological responses directly affected the function of the immune system. Positive emotional states could enhance the function of the immune system, while negative emotional states weaken the function of the immune system.^[^ ^14^ ^]^ Physiological and immune changes caused by depression are closely related to tumor recurrence and death, such as breast cancer patients with depression have a 30% higher risk of death than normal breast cancer, and the risk of recurrence is 24% higher than normal breast cancer.^[^ ^15^ ^]^

Cancer development is a dynamic process with many intrinsic factors contributing to its occurrence. The gut microbiota plays a crucial role in determining the fate of transformed cells. Microbial dysbiosis may promote tumorigenesis and lead to metastasis.^[^ ^16^ ^]^ Diet is a key determinant of the composition of the human gut microbiome. There is growing evidence that the effects of dietary intake on human health and disease are related to the immune system and the gut microbiota.^[^ ^17^ ^]^ Both short‐term and long‐term dietary changes affect the ecology of the gut microbiome. In turn, gut bacteria influence food cravings and eating behavior.^[^ ^18^ ^]^ These gut microbiotas affect the body's inflammatory response, stress resistance, neurological function, and even mental strength, all of which are relevant to exercise.^[^ ^18^, ^19^ ^]^

**Figure 2:**
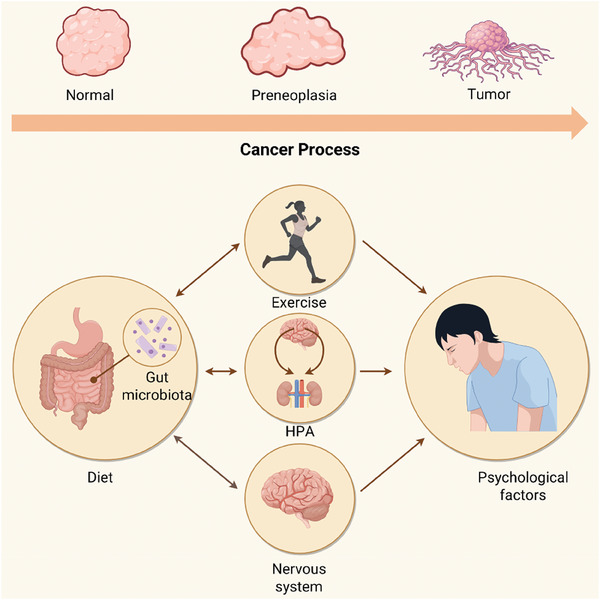
Person‐centered psychological factors, diet, and exercise on cancer.

Research has proved that gut microbiota dysbiosis will have an impact on mood, because many of the messenger substances (neurotransmitters, such as pentazocine, dopamine, etc.), and a variety of emotionally pleasurable hormones (e.g., serotonin), mainly synthesized in the intestine, and then by the “microbiota ‐gut‐brain axis” to the brain. Then it is delivered to the brain by the “colony‐gut‐brain axis”, forming the transmission of information. If the synthesis of these substances in the intestine decreases, the transmission of information will also decrease and mood will be affected. Metabolites of gut microbiota have also been shown to alter brain activity and exacerbate anxious behavior.^[^ ^20^ ^]^ Research has also found that gut microbiota affects the development of the hypothalamic‐pituitary‐adrenal (HPA) axis, and that dysfunction of the HPA axis may lead to mood disorders and abnormal stress responses.^[^ ^21^ ^]^ Studies have also found that changes in mood can affect the composition and function of gut microbiota. Negative emotions such as stress and anxiety may lead to disruption of the microbiota, decreasing the number of beneficial bacteria and increasing the growth of harmful bacteria. The above research suggests that the gut microbiota acts as a nexus, linking diet, exercise, and psychological factors to influence the onset and progression of cancer. Unhealthy nutrient intake structure, lack of exercise, and psychological depression are the factors for a higher risk of cancer and have a direct relationship with the occurrence of cancer (**Figure** **2**).

The above evidence showed that unhealthy nutrient intake structure, lack of exercise, and psychological depression were the factors for higher risk of cancer, and had a direct relationship with the occurrence of cancer. Unfortunately, most people have become accustomed to these bad habits and do not notice the potential cancer risks they bring. In this chapter, the relationship between psychology, nutrition, exercise, and cancer will be expounded in combination with previous studies and clinical data. It is expected to attract people's attention, consciously join the HHM system, and maintain good living habits and health concepts.

### 2.1 Psychological Factors

**Figure 3:**
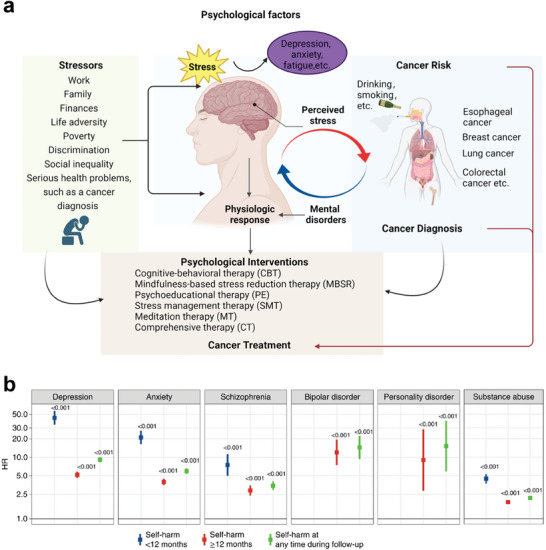
a) Impact of psychosocial factors and intervention on cancer‐relevant processes is illustrated. Psychological factors such as stress, anxiety, and depression have been proven to affect cancer incidence. b) The hazard ratio for risk of self‐harm for each psychiatric disorder was further adjusted for noncancer comorbidities, cancer treatment, and the presence of other psychiatric disorders.^[^ ^41^ ^]^ Copyright 2022, Springer Nature.

In today's world, a significant number of individuals experience psychological challenges as a result of various factors, including negative life events, work‐related stress, personality traits, and societal influences. These challenges may manifest as conditions like depression, anxiety, fatigue, feelings of helplessness, perceived pressure, distress, a stressed personality, unfavorable coping mechanisms, and a depressive mood.^[^ ^22^ ^]^ Chronic stress can endanger human health, which describes how people feel when they are under mental, physical, or emotional pressure. Sources of stress (factors that can cause stress) can come from people's daily responsibilities and routines, including work, family, and finances, as well as from other external factors, such as life adversity, poverty, discrimination, and social inequality. Serious health problems, such as a cancer diagnosis for oneself, a close friend, or a relative, can also cause stress (**Figure** **3**a).

People are concerned about whether stress can cause cancer.^[^ ^23^ ^]^ For example, a systematic evaluation and meta‐analysis of depression and anxiety concerning cancer incidence and mortality showed that clinically diagnosed depression and anxiety were associated with higher cancer incidence, poorer cancer survival, and higher cancer‐specific mortality.^[^ ^24^ ^]^ Nearly a quarter of women and a sixth of men have experienced depression in their lifetime, and up to 65% of individuals have recurrent episodes of the disorder.^[^ ^25^ ^]^ Chida et al. used a meta‐analysis method to assess the longitudinal relationship between stress‐related psychosocial factors and cancer. The results of 165 studies indicated that stress‐related psychosocial factors were associated with a higher cancer incidence in initially healthy populations (*p* = 0.005); depression increased the incidence of cancer by 29%, and stress‐related psychosocial factors increased the incidence of lung cancer by 23%.^[^ ^22b^ ^]^ Furthermore, depression in breast cancer patients predicted a 29% elevated risk for cancer‐specific mortality,^[^ ^26^ ^]^ and low levels of perceived social support, a smaller social network, being unmarried, or being depressed predicted a 12%–25% elevated relative risk for cancer mortality in various cancer types.^[^ ^27^ ^]^ Laboratory studies of animal and cancer cells have shown that chronic stress may lead to cancer progression and metastasis.^[^ ^28^ ^]^ Depression and anxiety may hinder cancer treatment and recovery, as well as quality of life and survival.^[^ ^29^ ^]^

In addition, a comprehensive study conducted in the United Kingdom involving 106 000 women revealed no conclusive evidence linking breast cancer risk to perceived stress levels or adverse life events within the past 5 years.^[^ ^30^ ^]^ Similarly, a 15‐year prospective study indicated that Australian women who were at an increased familial risk of breast cancer revealed that there was no observed connection between acute and chronic stressors, social support, optimism, or other emotional characteristics and the risk of developing breast cancer.^[^ ^31^ ^]^ In a comprehensive meta‐analysis conducted in 2019, nine observational studies from Europe and North America were examined. A notable correlation between work stress and esophageal cancer was discovered in Europe, while no such link was observed in North America. Their findings suggest that job stress is a significant risk factor for colorectal, lung, and esophageal cancers. However, no association was observed between work stress and the risk of prostate, breast, or ovarian cancer.^[^ ^32^ ^]^ Likewise, a meta‐analysis of 12 European cohort studies revealed that there is a discernible link between work‐related stress and the likelihood of developing lung, colorectal, breast, or prostate cancer.^[^ ^33^ ^]^ The potential impact of stress on cancer development is still a topic of debate. There is a correlation between stress and an increased risk of cancer. Individuals who experience chronic stress may adopt unhealthy habits like smoking, overeating, lack of exercise, or excessive alcohol consumption, all of which are linked to a higher likelihood of developing certain types of cancer.

Cancer diagnosis can also have a significant impact on mental health and well‐being. Common emotions during this life‐changing experience include anxiety, distress, and depression. Research has shown that ≈50% of cancer patients experience a mental illness, such as clinically significant emotional distress and/or unrecognized or untreated psychosocial conditions, as a result of cancer at some point during the cancer trajectory.^[^ ^34^ ^]^ Studies have shown that psychological distress may be prevalent among 6%–75% of cancer patients, depending on the number of patients and measurement tools, and varies by instrument type, cancer type, and treatment stage.^[^ ^35^ ^]^ Cancer‐related fatigue was present in 91% of breast cancer patients, which had severe negative consequences for quality of life and daily activities.^[^ ^36^ ^]^ In addition, young cancer survivors and female survivors of working age seem to be more susceptible to depression and anxiety.^[^ ^37^ ^]^ To help individuals who have been affected by cancer, it is widely recognized that a comprehensive approach to addressing the physical, emotional, and social challenges is crucial. At present, the impact of psychological education programs on psychological stress and the quality of life of cancer survivors has received increasing attention. Studies have shown that psychological education can reduce distress, anxiety, depression, and anger and improve interpersonal relationships, role performance, sexual life, and quality of life.^[^ ^38^ ^]^ A cohort study revealed that individuals with cervical cancer who had a pre‐existing psychiatric disorder experienced lower overall survival rates and cervical cancer‐specific survival rates compared to those without a prior psychiatric diagnosis.^[^ ^39^ ^]^ A cross‐sectional study of 7509 cancer outpatients (51.4% women) demonstrates that about one‐third of the sample showed symptoms of anxiety (35.2%) or depression (27.9%), and one in six patients were likely to have a mental illness, with women more likely to be affected. The prevalence of anxiety and depression was significantly higher in patients with lung and brain cancer than in patients with other cancers.^[^ ^40^ ^]^

A study estimated the risk of self‐harm in 459542 cancer patients following a diagnosis of a mental disorder and the risk of wrongful death following self‐harm. Patients undergoing chemotherapy, radiotherapy, and surgery experienced the highest cumulative burden of mental disorders. The burden of mental disorders was highest in patients receiving alkylating chemotherapy and lowest in those receiving kinase inhibitor therapy. All psychiatric disorders were associated with an increased risk of subsequent self‐harm, with the highest risk observed within 12 months of psychiatric diagnosis. Patients who self‐harm are 6.8 times more likely to die of non‐natural causes within 12 months of self‐harm compared to controls (Figure 3b).^[^ ^41^ ^]^

Psychological interventions, such as positive thought stress reduction, have also been shown to effectively reduce psychological stress and improve quality of life. Positive thought‐based interventions, such as positive thought stress reduction and positive thought cognitive therapy, have also been studied in cancer care. These interventions help patients develop positive thinking skills, such as focusing on the present moment without judgment, to reduce stress and improve mental health. Studies have shown that positive thinking interventions can be effective in reducing anxiety, depression, and fatigue in cancer patients. A network meta‐analysis of six different psychosocial interventions including cognitive‐behavioral therapy, mindfulness‐based stress reduction therapy (MBSR), psychoeducational therapy, stress management therapy, meditation therapy, and comprehensive therapy, showed that MBSR was most likely to be the best psychosocial intervention to relieve cancer patients.^[^ ^42^ ^]^

Additional psychological interventions that have been researched in cancer care involve relaxation techniques, such as progressive muscle relaxation and guided imagery, as well as supportive counseling. Although the evidence for these interventions varies, they have the potential to help certain patients by reducing psychological distress and enhancing their quality of life. A study examining the effects of home‐based psychological education programs on breast cancer survivors found that after 6 months of intervention, the quality of life of these survivors significantly improved compared to the initial baseline. This suggests that psychological education programs can be effective in reducing distress, anxiety, and depression, and enhancing the overall well‐being of breast cancer survivors.^[^ ^43^ ^]^

Overall, the significance of psychology in cancer care is being increasingly acknowledged as a crucial element of HHM. Addressing the psychological needs of cancer patients can not only improve their quality of life but may also improve treatment outcomes. More research is needed to identify the most effective psychological interventions for different patient populations and to understand better the mechanisms underlying their effects. In summary, psychological factors can significantly impact cancer patients' physical health and treatment outcomes. Addressing psychological needs has become an important aspect of HHM in cancer care. Cognitive‐behavioral therapy, mindfulness‐based interventions, and other psychological approaches can be effective in reducing symptoms of anxiety and depression and improving the quality of life among cancer patients. More research is needed to identify the most effective psychological interventions for different patient populations and to understand better the mechanisms underlying their effects.

### 2.2 Nutrition

**Figure 4:**
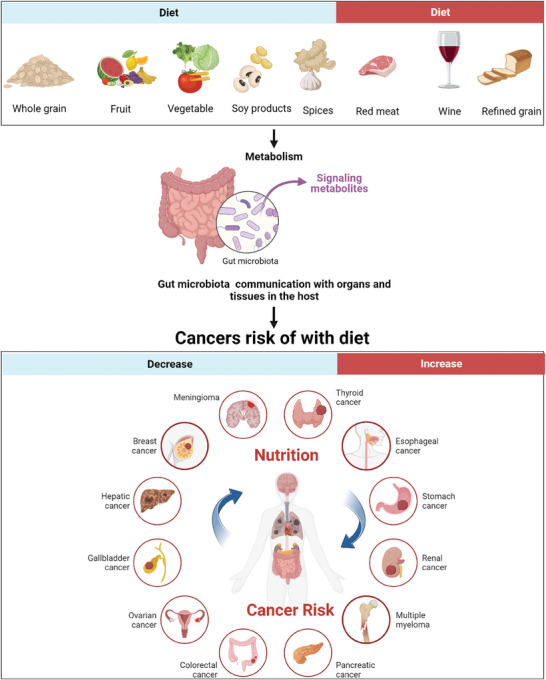
A diet rich in whole grains, fruits, vegetables, spices, and soy products can Reduce the risk of many types of cancer while red meat, wine, and refined grain intake will increase.

Nutrition is an essential component of cancer treatment and recovery. A well‐balanced diet can help strengthen the immune system, improve energy levels, and reduce the risk of side effects from treatment. A diet rich in fruits, vegetables, whole grains, and lean protein sources can provide the necessary nutrients to support the body during therapy through gut microbiota that can communicate with organs and tissues in the host (**Figure** **4**). Grains like rice, barley, wheat, millet, corn, sorghum, oats, and buckwheat, among others., are rich sources of essential nutrients such as proteins, fats, minerals, vitamins, and fibers.^[^ ^44^ ^]^

Several epidemiological studies have indicated that grain intake is negatively correlated with cancer incidence rates. Research has found a significant link between consuming whole grains and a reduced risk of breast cancer. One study revealed a 16% decrease in risk, while another study showed even more promising results, with higher intakes of whole grains associated with a 39% and 47% lower risk of breast cancer.^[^ ^45^ ^]^ Without exception, several meta‐analyses reported significant reductions in colorectal cancer risk associated with whole grain intake, showing an 11% to 21% lower risk of colorectal cancer.^[^ ^46^ ^]^ A meta‐analysis of the relationship between whole grain intake and the risk of gastric cancer has been published (ranging from 13% to 50% lower risk for the highest whole grain intake).^[^ ^47^ ^]^ Both retrospective and prospective studies have reported a reduction in the risk of high whole grain intake with a 35%–60% reduction of esophageal cancer.^[^ ^48^ ^]^ Two meta‐analyses have shown 24% and 30% lower risk of pancreatic cancer.^[^ ^47^, ^49^ ^]^

Vegetables contain various bioactive phytochemicals, including glucosinolates, isothiocyanates, and carotenoids. These compounds are highly regarded for their potential to prevent cancer and other chronic diseases.^[^ ^50^ ^]^ Liu et al. conducted a meta‐analysis of 13 epidemiological studies (11 case‐control studies and 2 cohort studies); the combined results of all studies showed that high cruciferous vegetable intake was significantly associated with reduced breast cancer risk (R2 = 0.85, 95% CI, 0.77–0.94).^[^ ^51^ ^]^ A meta‐analysis of 72 different studies reported that lycopene intake and serum lycopene levels were negatively correlated with various cancers, such as breast, prostate, cervical, ovarian, liver, and other organ cancers. Since then, multiple studies have reported that with increased lycopene intake and serum levels of lycopene, the risk of cancer has been reduced significantly.^[^ ^52^ ^]^ Studies by Giovannucci et al. showed that daily consumption of lycopene‐rich foods was associated with a 30%–40% reduction in prostate cancer risk.^[^ ^53^ ^]^ And research demonstrated that lycopene, which is mainly supplied by tomatoes, was associated with a 31% lower risk of pancreatic cancer in men.^[^ ^54^ ^]^

Fruits often contain a high polyphenols content, which gives them great antioxidant activity and may help reduce the risk of cancer.^[^ ^55^ ^]^ Grape and its processed products such as wine, are widely consumed worldwide all over the world and are recognized as healthy food. Anthocyanins, flavonoids, and resveratrol are abundant in grape skins, seeds, and red wine, which are considered to have strong antioxidant, anticancer, and anti‐inflammatory properties.^[^ ^56^ ^]^ Several reviews and meta‐analyses of observational studies show that dietary flavonoids (total or individual) are associated with reduced risk of breast,^[^ ^57^ ^]^ ovarian,^[^ ^58^ ^]^ esophageal,^[^ ^59^ ^]^ gastric^[^ ^60^ ^],^ and lung cancer^[^ ^61^ ^]^. Wang et al. conducted a meta‐analysis of seven studies, which indicates a significant inverse association between total anthocyanin intake and colorectal cancer risk (R^2^ = 0.78; 95% CI, 0.64–0.95). Likewise, there was significant evidence of a relationship between anthocyanin intake and colorectal cancer in the colon site (R^2 ^= 0.81; 95% CI, 0.71–0.92).^[^ ^62^ ^]^ In addition, resveratrol is a naturally occurring polyphenolic phytoalexin. Limagne et al. found that resveratrol interferes with proinflammatory signal pathways triggered by IL1‐β leading to inhibition of inflammation, which is often involved in cancer onset and progression by regulating proliferation, apoptotic cell death, and angiogenesis.^[^ ^63^ ^]^ Apples (Malus pumila) are widely known healthy fruits. Because it is rich in bioactive phytochemicals (phenols and flavonoids), it has strong chemopreventive and antioxidant activity,^[^ ^64^ ^]^ which can reduce the risk of disease. Using the random‐effect model, Hyson et al. found that the high intake of apples was significantly associated with a reduced risk of lung cancer in both case‐control (OR = 0.75; 95% CI, 0.63–0.88; *p* = 0.001, I^2^ = 0%; and cohort (R^2^ = 0.89; 95% CI, 0.84–0.94; *p* < 0.001, I^2 ^= 68%) studies. A significant reduction in colorectal cancer risk associated with apple intake was observed in the case‐control (OR = 0.56; 95% CI, 0.54–0.81; *p* < 0.001, I^2^ = 55%). Further, they found that when data from case‐control studies were combined with data from cohort studies, meta‐analysis showed a significant reduction in the risk of lung (12%), colorectal (28%), oesophageal (34%), digestive tract (41%) and breast (11%) cancer.^[^ ^65^ ^]^ Limonoid is a rich bioactive component in citrus fruits such as oranges, lemons, grapefruit, pomelo, and lime, which has significant anti‐cancer effects. Bae et al. conducted a meta‐analysis to assess whether citrus fruit intake was associated with gastric cancer risk. The results showed that the incidence rate of gastric cancer decreased by 13% according to the intake of citrus fruits (sES, 0.87; 95% CI, 0.76–0.99; I2 = 69.6%). In subgroup analysis, citrus fruit intake inhibited cardia gastric cancer (CGC) (sES, 0.67; 95% CI, 0.55–0.81; I2 = 46.1%), and citrus fruit intake of 100 g per day inhibited CGC by 40% (R2 = 0.60; 95% CI, 0.44–0.83).^[^ ^66^ ^]^ In a meta‐analysis of an observational study, there was a significant inverse correlation between citrus fruit intake and bladder cancer risk in all pooled studies (R2 = 0.85; 95% CI, 0.76–0.94), and the results showed that citrus fruit intake was related to the reduction of bladder cancer risk.^[^ ^67^ ^]^ In a quantitative systematic review, the risk of breast cancer associated with high intakes of citrus fruits was reduced by 10% (OR = 0.90; 95% CI, 0.85–0.96; *p* < 0.001); results were consistent across the studies (I2 = 0).^[^ ^68^ ^]^ Another quantitative systematic review of citrus fruit intake and pancreatic cancer risk suggested an inverse association in the risk of pancreatic cancer with citrus fruit intake (OR = 0.83; 95% CI, 0.70–0.98).^[^ ^69^ ^]^ Mangosteen is known as the “queen fruit” in mangosteen‐rich flavonoid substances and has a variety of bioactivities, such as antioxidant, anti‐inflammatory, anticancer, and so on^[^ ^70^ ^]^. α‐Mangostin, the most abundant xanthone derived from the pericarps of mangosteen (78% content), is one of the most studied chemical preventive phytochemicals. α‐mangostin has many pharmacological activities, such as antioxidant, anti‐infective, anticarcinogenic, and antidiabetic activities, as well as neuroprotective, hepatoprotective, and cardioprotective properties, among which anticancer activity is the most promising.^[^ ^11^, ^71^ ^]^ A great deal of evidence from in vitro and in vivo studies demonstrated that α‐mangostin is suitable for all the major stages of tumor growth: initiation, promotion, and progression. In addition, α‐mangostin has been shown to modulate the concentration of major cell cycle mediators in the micromolar level, causing a blockage of the G1/S transition, leading to G1‐phase cell cycle arrest in many cancers, such as prostate cancer, melanoma, breast cancer, pancreatic cancer.^[^ ^72^ ^]^

Consuming a healthy diet has been shown to enhance the physical and psychosocial health of cancer survivors and reduce the risk of cancer recurrence, cancer specificity, and all‐cause mortality.^[^ ^73^ ^]^ Pekmezi et al. summarized 21 randomized controlled trials, and the results showed that dietary intervention could improve the quality of diet, nutrition‐related biomarkers, and body weight. In addition, diet may positively affect biomarkers related to progressive diseases and overall survival (such as insulin level, DNA oxidative damage, and tumor proliferation rate), which provides evidence to support dietary interventions for cancer survivors.^[^ ^74^ ^]^ It is estimated that increasing individual consumption of fruits and vegetables to up to 600 g per day will reduce the total burden of disease worldwide by 1.8%, with potential reductions of 19%, 20%, 12%, and 2% for stomach, oesophageal, lung and colorectal cancers, respectively.^[^ ^75^ ^]^ Lee et al. found that dietary intervention significantly increased the probability of achieving the goal of reducing red meat and processed meat consumption and refined grains in the 6th and 24th month, and the proportion of colorectal cancer survivors significantly increased with the significant reduction of red meat and processed meat and refined grains intake.^[^ ^76^ ^]^ Antwi et al. found that the increase of the percentage of α‐tocopherol, trans‐β‐carotene, β‐cryptoxanthin, cis‐lutein/zeaxanthin, and all‐trans‐lycopene levels was related to the decrease of prostate‐specific antigen (PSA) level, suggesting that greater intake of foods containing these micronutrients might be beneficial to men with PSA‐defined prostate cancer recurrence.^[^ ^77^ ^]^ In addition, the ACS guidelines for nutrition and physical activity for cancer survivors recommend that prostate cancer survivors should emphasize vegetables and fruits rich in micronutrients and phytochemicals, low amounts of saturated fat, at least 600 IU of vitamin D per day, and adequate but not excessive dietary sources of calcium (i.e., no more than 1200 mg day^−1^).^[^ ^78^ ^]^

Soy products have been widely consumed in Asian regions as a common food in diets. Many of the potential health benefits are associated with the intake of soy products, such as reduced incidence of breast cancer,^[^ ^79^ ^]^ lung cancer,^[^ ^80^ ^]^ and colon cancer.^[^ ^81^ ^]^ Soy products are rich in isoflavones, and a meta‐analysis of prospective studies indicated that intake of isoflavones was significantly associated with reduced risk of lung and gastric cancer and nearly significant breast and colorectal cancers.^[^ ^82^ ^]^ In addition, soybean contains many bioactive components against colon cancer, such as isoflavones, saponins, polypeptides, soluble polysaccharides,^[^ ^81^ ^]^ and a high proportion of non‐digested carbohydrates that were fermented in the colon to produce short‐chain fatty acids which elicited protective effects against colon cancer by modulating protein expression in HT‐29 cells.^[^ ^83^ ^]^ Additionally, legumes are rich in bioactive nutrients (phytosterols, copolymers, polyphenols, triterpenic acids) that have shown a certain degree of correlation with cancer prevention.^[^ ^84^ ^]^ Moreover, soy has been shown to contain large amounts of plant protein, and high levels of potassium, magnesium, folic acid, selenium, and phosphorus, which are of great benefit to human health.^[^ ^85^ ^]^

Spices are widely used in folk medicine and food seasoning. In recent years, many studies have shown that curcumin in turmeric, eugenol in clove, capsaicin in pepper, and allicin in garlic and other spices and their bioactive components have significant antioxidant, anti‐inflammatory, and antiproliferation effects, showing the potential to prevent and treat cancer.^[^ ^86^ ^]^ Garlic (Allium sativum L.) is a common spice that has many kinds of health benefits, mainly because it has many different types of bioactive compounds, such as organic sulfide, saponin, phenolic compound, polysaccharide, etc., and it is a typical food, which has a long history as a traditional medicine in China. Many studies have shown that garlic and its active constituents can prevent various cancers, such as colorectal, lung, gastric, and bladder cancer.^[^ ^87^ ^]^ Pourzand et al. conducted a case‐control study, which suggested that high consumption of certain Allium vegetables, especially garlic, could reduce the risk of breast cancer, with an adjusted ORs of 0.41 (95% CI, 0.20–0.83).^[^ ^88^ ^]^ Kodali et al. conducted a meta‐analysis to determine whether garlic intake reduced the risk of gastric cancer. A meta‐analysis of a total of 8621 cases and 14889 controls was conducted. The results showed that high, low, and any garlic intake were related to the risk reduction of gastric cancer, among which high intake had the most significant risk reduction, (OR = 0.49, 95% CI, 0.38–0.62).^[^ ^89^ ^]^ Galeone et al. found that garlic can prevent colorectal cancer. Compared with low or no garlic use (p < 0.001), intermediate and high garlic use were associated with decreased risk of colorectal cancer, with ORs of 0.88 (95% CI, 0.78–0.98) and 0.74 (95% CI, 0.63–8.86), respectively.^[^ ^90^ ^]^

Moreover, cancer treatment can cause changes in appetite, taste, and digestion, affecting nutritional status. Therefore, it is crucial to work with a registered dietitian to develop an individualized nutrition plan that meets each patient's specific needs. Nutrition plays a vital role in cancer care, both in terms of preventing cancer and managing cancer‐related symptoms and treatment side effects. A healthy diet can help patients maintain their strength, manage treatment side effects, and potentially reduce the risk of cancer recurrence.^[^ ^91^ ^]^ Research has shown that certain diet types may benefit cancer patients more than others.

1. Mediterranean diet: This diet emphasizes fruits, vegetables, whole grains, lean proteins, and healthy fats such as olive oil. Studies have found that a Mediterranean diet may help reduce the risk of several types of cancer, including breast cancer, colon cancer, and prostate cancer. Additionally, a Mediterranean diet may help improve treatment outcomes and reduce side effects in cancer patients.
2. Plant‐based diet: This diet focuses on fruits, vegetables, whole grains, legumes, and nuts, and may include limited amounts of lean protein such as fish or poultry. Studies have suggested that a plant‐based diet may help reduce the risk of certain cancers, including breast, colon, and prostate cancer, and may improve treatment outcomes in cancer patients.
3. Ketogenic diet: This diet is low in carbohydrates and high in fat, which forces the body to use fat for energy instead of glucose. Some studies have suggested that a ketogenic diet may help improve treatment outcomes in cancer patients, but the evidence is still limited and more research is needed.
4. Low‐fat diet: This diet emphasizes fruits, vegetables, whole grains, and lean protein sources such as fish or poultry, and restricts high‐fat foods such as red meat, cheese, and butter. Studies have suggested that a low‐fat diet may help reduce the risk of certain cancers, including breast cancer, and may improve treatment outcomes in cancer patients.

It is important to note that every patient's nutritional needs are unique, and a registered dietitian can help develop an individualized plan based on their specific diagnosis, treatment plan, and personal preferences. It is also important to consider any side effects of cancer treatment that may impact a patient's ability to eat or tolerate certain foods, such as nausea, vomiting, or mouth sores. In conclusion, a healthy diet can play a critical role in cancer care, helping to improve treatment outcomes, manage side effects, and potentially reduce the risk of cancer recurrence. Although more researches are needed to determine the efficacy of specific diets in cancer care, a diet that emphasizes whole, nutrient‐dense foods and limits processed and high‐fat foods is generally recommended for cancer patients.

Cancer patients often experience a variety of symptoms and side effects related to their disease and treatment, such as fatigue, nausea, vomiting, loss of appetite, and weight loss. Adequate nutrition is essential to help patients maintain their strength, support their immune system, and manage these symptoms. There are several ways in which nutrition can impact cancer patients' health and treatment outcomes:

1. Weight management: Maintaining a healthy weight is important for cancer patients as obesity is a risk factor for several types of cancer, and weight gain during treatment may worsen treatment outcomes. A balanced diet that is rich in fruits, vegetables, whole grains, and lean proteins can help patients maintain a healthy weight.
2. Immune function: Cancer and its treatment can weaken the immune system, making patients more vulnerable to infections. Nutrients such as protein, vitamin C, vitamin D, and zinc are important for immune function, and a balanced diet can help support the immune system.
3. Treatment side effects: Chemotherapy, radiation therapy, and other cancer treatments can cause side effects such as nausea, vomiting, and diarrhea, affecting a patient's ability to eat and absorb nutrients. Certain foods and dietary strategies, such as eating small, frequent meals, avoiding spicy or greasy foods, and staying hydrated, can help manage these symptoms.
4. Cancer prevention: While a healthy diet cannot guarantee that a person will not develop cancer, evidence suggests that specific dietary patterns can help reduce the risk of certain types of cancer. For example, a diet that is rich in fruits, vegetables, whole grains, lean proteins and low in processed and high‐fat foods may help reduce the risk of breast, colon, and prostate cancer.
5. Cancer recurrence: After completing cancer treatment, patients may be concerned about the risk of cancer recurrence. While there is no guaranteed way to prevent cancer from returning, a healthy diet and lifestyle can help reduce the risk. For example, maintaining a healthy weight, engaging in regular physical activity, and consuming a diet that is rich in fruits, vegetables, whole grains, and lean proteins can help reduce the risk of cancer recurrence.

In summary, nutrition plays a critical role in cancer care and several types of diets of diets that have been about cancer. While more research is needed to determine the efficacy of specific diets in cancer care, a balanced diet that is rich in whole, nutrient‐dense foods and limited in processed and high‐fat foods is generally recommended for cancer patients. A registered dietitian can help develop individualized plans based on a patient's needs and preferences. New nutrition guidelines for cancer survivors suggested: that breast cancer survivors should avoid obesity, diets should include low amounts of saturated fat, but high amounts of vegetables and fruits, a moderate amount of soy products, and a moderate or non‐alcoholic diet; lung cancer survivors are recommended to take multivitamin/mineral supplements, small, frequent meals that are concentrated in calories and easy to swallow are most beneficial; esophageal cancer survivors should eat high protein, low fat, high carbohydrate food, avoid chocolate, fat, alcohol, coffee, spearmint, peppermint, garlic, and onion; colorectal cancer survivors should adjust their diet to adapt to the interruption of cancer survivors should use sugar‐free gum and mint, oral rinses and gels can relieve symptoms and increase appetite, and liquid, pureed or juice food may be the first choice.

### 2.3 Exercise

Exercise is an important component of cancer care and can provide several benefits to cancer patients, both during and after treatment. Physical activity has attracted attention due to the rapid accumulation of evidence of its protective effect. In March 2018, the American College of Sports Medicine convened the second round‐table conference on Exercise and Cancer Prevention and Control, presenting evidence that exercise is associated with reduced risk of developing cancer and improved survival after cancer diagnosis.^[^ ^92^ ^]^

Moore et al. pooled a prospective cohort of 12 European and U.S. studies of the association between physical activity and cancer risk, including 1.44 million participants and 186932 cancer cases. This meta‐analysis concluded that both high versus low levels of physical activity during leisure time were associated with a lower risk of 13 cancers: esophageal adenocarcinoma (HR, 0.58; 95% CI, 0.37–0.89), liver (HR, 0.73; 95% CI, 0.55–0.98), lung (HR, 0.74; 95% CI, 0.71–0.77), kidney (HR, 0.77; 95% CI, 0.70–0.85), gastric cardia (HR, 0.78; 95% CI, 0.64–0.95), endometrial (HR, 0.79; 95% CI, 0.68–0.92), myeloid leukemia (HR, 0.80; 95% CI, 0.70–0.92), myeloma (HR, 0.83; 95% CI, 0.72–0.95), colon (HR, 0.84; 95% CI, 0.77–0.91), head and neck (HR, 0.85; 95% CI, 0.78–0.93), rectal (HR, 0.87; 95% CI, 0.80‐0.95), bladder (HR, 0.87; 95% CI, 0.82–0.92), and breast (HR, 0.90; 95% CI, 0.87–0.93).^[^ ^93^ ^]^

Exercise can reduce the risk of developing a primary cancer and its possible biological mechanisms include changes in endogenous sexual and metabolic hormone levels, growth factors, reduction of obesity and central obesity, and possible changes in immune function. Obesity is one of the most serious public health problems in the world. Numerous epidemiological studies have shown that obesity is associated with an increased risk of various cancers, including colon, endometrial, postmenopausal breast, kidney, esophageal, pancreatic, gallbladder, liver, and hematological malignancies.^[^ ^94^ ^]^ The potential biological mechanisms between obesity and cancer include hormones, growth factors, modulation of energy balance and calorie restriction, multiple signaling pathways, and inflammatory processes related to obesity.^[^ ^95^ ^]^ Therefore, weight control and maintaining a healthy weight may be a particularly important pathway of the biological mechanism linking physical activity and cancer prevention, and one of the most important methods for preventing cancer. In addition, physical activity may have an impact on the occurrence of cancer through the reduction of obesity.

It can provide patients with several physical and psychological benefits during and after treatment. Regular physical activity can help cancer patients maintain their strength, manage treatment‐related side effects, and potentially reduce the risk of cancer recurrence. Regular physical activity has been shown to improve quality of life, reduce fatigue and depression, and enhance immune function. Exercise can also help manage the side effects of cancer treatment, such as lymphedema and weight gain. However, it is important to work with a healthcare professional to develop a safe exercise plan that takes into account any physical limitations or restrictions due to cancer treatment. For example, some cancer treatments may weaken bones, making certain types of exercise unsafe.

There are several ways in which exercise can impact cancer patients' health and treatment outcomes:

1. Improving physical function: Cancer and its treatment can cause fatigue, weakness, and reduced physical function. Exercise can help improve patients' strength, endurance, and overall physical function. Studies have shown that exercise can improve cardiorespiratory fitness, muscle strength, and quality of life in cancer patients.
2. Reducing treatment‐related side effects: Cancer treatments such as chemotherapy and radiation therapy can cause side effects such as fatigue, nausea, and neuropathy. Exercise can help manage these side effects by reducing fatigue, improving appetite, and reducing treatment‐related inflammation.
3. Reducing the risk of recurrence: There is evidence to suggest that regular physical activity may help reduce the risk of cancer recurrence. Studies have shown that exercise can improve immune function, reduce inflammation, and help regulate hormones such as insulin and estrogen, all of which may contribute to a reduced risk of cancer recurrence.
4. Managing comorbid conditions: Cancer patients may have other health conditions such as diabetes or heart disease. Exercise can help manage these conditions by improving blood sugar control, reducing blood pressure, and improving cardiovascular fitness.

Several types of exercises have been studied concerning cancer care as shown in **Table** **2**.

**Table 2:** The types of exercise concerning cancer care.

Type	Description and features	Influences on cancer patients
Aerobic exercise	increase heart rate and breathing rate, such as brisk walking, cycling, or swimming	Aerobic exercise can improve cardiorespiratory fitness, reduce fatigue, and improve quality of life in cancer patients.
Resistance training	lifting weights or using resistance bands to build muscle strength	Resistance training can improve muscle strength, reduce fatigue, and improve quality of life in cancer patients.
Yoga	combination of physical postures, breathing techniques, and meditation	Yoga can improve physical function, reduce fatigue, and improve quality of life in cancer patients.
Tai Chi	slow, flowing movements and deep breathing	Tai chi can improve balance, reduce falls, and improve quality of life in cancer patients.

In addition, exercise has been shown to have a positive impact on mental health and well‐being. Cancer patients may experience anxiety, depression, and other psychological symptoms as a result of their diagnosis and treatment. Exercise can help reduce these symptoms by increasing endorphin levels, promoting relaxation, and improving self‐esteem. Several types of exercise have been studied concerning cancer care:

1. Aerobic exercise: This type of exercise involves activities that increase heart rate and breathing rate, such as brisk walking, cycling, or swimming. Studies have shown that aerobic exercise can improve cardiorespiratory fitness, reduce fatigue, and improve the quality of life in cancer patients.
2. Resistance training: This type of exercise involves lifting weights or using resistance bands to build muscle strength. Studies have shown that resistance training can improve muscle strength, reduce fatigue, and improve quality of life in cancer patients.
3. Yoga: This type of exercise involves a combination of physical postures, breathing techniques, and meditation. Studies have shown that yoga can improve physical function, reduce fatigue, and improve quality of life in cancer patients.
4. Tai Chi: This type of exercise involves slow, flowing movements and deep breathing. Studies have shown that tai chi can improve balance, reduce falls, and improve quality of life in cancer patients.

In conclusion, exercise is important in cancer care, providing several benefits to patients during and after treatment. Different types of exercise have been studied in relation to cancer care, and a combination of aerobic exercise, resistance training, and mind‐body exercises such as yoga and tai chi may be beneficial for cancer patients. Cancer patients need to talk to their healthcare team before starting an exercise program to ensure that it is safe and appropriate for their individual needs. Cancer patients need to talk to their healthcare team before starting an exercise program to ensure that it is safe and appropriate for their individual needs. In some cases, modifications may be needed to accommodate a patient's medical condition or treatment‐related side effects. However, with proper guidance and supervision, exercise can be a safe and effective way for cancer patients to improve their physical and psychological well‐being.

Cancer survivors often expect their work or daily life to return to a level similar to before the cancer diagnosis. While cancer therapy has been shown to be effective in prolonging survival, it can lead to increased fatigue, decreased physical activity, and reduced quality of life. The side effects of these therapies may prolong and hinder the patient's return to everyday life.^[^ ^96^ ^]^ In addition, cancer survivors may lose their skeletal muscle strength due to cancer or problems left over by cancer treatment.^[^ ^97^ ^]^ However, randomized controlled trials show that exercise intervention can improve skeletal muscle strength and restore the physical fitness of cancer survivors.^[^ ^98^ ^]^ Physical activity is a potentially attractive intervention, which can reduce cancer‐related sequelae and help patients return to the health status they had before treatment. In 2008, the US Department of Health and Human Services issued guidelines for American sports activities, which stated that when individuals with chronic diseases (such as cancer) cannot meet the recommendations based on their health status, they “should be as physically active as their abilities and conditions allow”. An explicit recommendation was made to “avoid inactivity” and make it clear that “some physical activity is better than none”.^[^ ^99^ ^]^ Fong et al. conducted a meta‐analysis consisting of 34 randomized controlled trials, 22 of which (65%) focused on breast cancer patients, based on the study of breast cancer patients, physical activity was associated with improvements in insulin‐like growth factor‐I, bench press, leg press, fatigue, depression, and quality of life and when combined with different types of cancer (prostate, gynecological, colorectal, gastric and lung cancer), it was found that body mass index, body weight, peak oxygen consumption, peak power output, 6‐min walking distance, right‐hand grip strength and quality of life were significantly improved.^[^ ^96^ ^]^ Sheehan et al found that exercise training intervention increased cognitive function, overall quality of life, and functional fitness of cancer survivors, reduced insomnia and fear of physical activity, and greatly alleviated cancer fatigue.^[^ ^100^ ^]^

Observational studies indicate that long‐term and moderate‐intensity aerobic exercise reduces the visceral adipose tissue content, increases health‐related fitness, and reduces the recurrence risk of colon cancer among patients with Stages I, II, and III disease improves the favorable prognosis of biomarkers and the multiple health‐related quality of life outcomes of patients with colon cancer.^[^ ^101^ ^]^

In addition, anticancer treatment can produce many side effects, including chronic non‐soluble inflammation and reduced immune function, reducing the overall quality of life.^[^ ^102^ ^]^ Exercise training is a non‐pharmacological complementary therapy, which helps symptom management by systematically targeting specific adverse effects of treatment, including inflammation,^[^ ^103^ ^]^ and can also enhance immune function. Khosravi et al. found that combined aerobic and resistance training reduced the pro‐inflammatory markers, C‐reactive protein, and tumor necrosis factor were the most sensitive to changes, and exercise training tended to reduce anti‐inflammatory markers and improve immunity.^[^ ^104^ ^]^

## 3 Mechanisms of Psychology, Nutrition, and Exercise in Regulating Cancer

The occurrence and development of tumors are influenced by many factors. Among them, psychological, nutritional, and exercise factors are closely related to the occurrence of tumors. Research indicates that psychological factors such as stress, anxiety, and depression increase the risk of cancer. Prolonged psychological stress can lead to a decline in immune system function, hormonal imbalances, and enhanced inflammation, thereby increasing the risk of cancer.^[^ ^105^ ^]^ In addition, some studies have found that psychological interventions can improve patient quality of life and treatment outcomes.^[^ ^106^ ^]^ Malnutrition is an important risk factor for the development of tumors.^[^ ^107^ ^]^ High‐fat, high‐sugar, and high‐salt diets increase the risk of cancer. Conversely, a balanced diet structure and moderate exercise can improve the body's immune system and reduce the risk of cancer. Furthermore, some studies have shown that certain nutrients such as vitamins C and D have anti‐cancer effects.^[^ ^108^ ^]^ Moderate exercise can improve the body's immune system and reduce the risk of cancer. Studies have shown that long‐term adherence to aerobic exercise can reduce the occurrence of chronic diseases such as obesity and cardiovascular disease, while also reducing the risk of cancer.^[^ ^109^ ^]^ Additionally, some studies have found that exercise can promote apoptosis and autophagy of tumor cells, thereby inhibiting tumor growth and metastasis.^[^ ^110^ ^]^ In summary, psychology, nutrition, and exercise regulate tumor development through various mechanisms and signaling pathways. This chapter will provide a detailed exploration of their tumor‐regulating mechanisms, aiming to illustrate the relationships between these three factors and cancer development at the molecular level.

### 3.1 Psychological Factors

Immune‐related psychological factors are considered potential contributors to cancer initiation and progression, as the immune system plays a crucial role in tumor surveillance and prevention of tumor progression and metastatic spread. There is substantial evidence that psychological factors can influence cellular and humoral indicators' immune status and function.^[^ ^111^ ^]^ The HPA axis may not work properly when someone is depressed or under a lot of stress. This can weaken their immune system and make some types of cancer more likely to spread. In general, both stressors and depression are associated with reduced cytotoxic T‐cell and natural killer (NK) cell activity, which influence processes such as immune surveillance of tumors, and events that regulate the development and accumulation of somatic mutations and genomic instability.^[^ ^112^ ^]^

Physiological and psychological stressors include external stimuli from the central nervous system (CNS), and ongoing CNS‐derived processes (e.g., anxiety and rumination about cancer) are sensed and processed by the CNS and trigger stress responses. Thus, the pituitary gland releases endorphins, oxytocin, prolactin, vasopressin, adrenocorticotropic hormone (ACTH), and other stress mediators, and activation of the HPA axis through hypothalamic corticotropin‐releasing hormone and systemic ACTH release leads to secretion of glucocorticoids (for example, cortisol) from the adrenal cortex. At the same time, the CNS activates the sympathetic nervous system (SNS), resulting in the secretion of adrenergic factors from the adrenal medulla (primarily adrenaline) and sympathetic nerve endings (mainly norepinephrine). These stressors contribute to most features of cancer by affecting malignant tissue, its microenvironment, immunity, lymphatic flow, and distant potential pre‐metastatic niches. Malignant tissues can contribute to stress responses (e.g., through interleukin‐6 (IL‐6) and IL‐1β) through local and systemic inflammation, affect the CNS, dysregulate HPA axis activity, and promote depression, sleep disturbances, and cancer‐associated fatigue. Overall, CNS‐initiated stress responses may exacerbate tumor growth and spread, as well as peripheral stress inflammatory cytokine responses, which feed back to the CNS to alter cognition and mood, promote stress responses, and create a vicious circle.^[^ ^28^ ^]^

**Figure 5:**
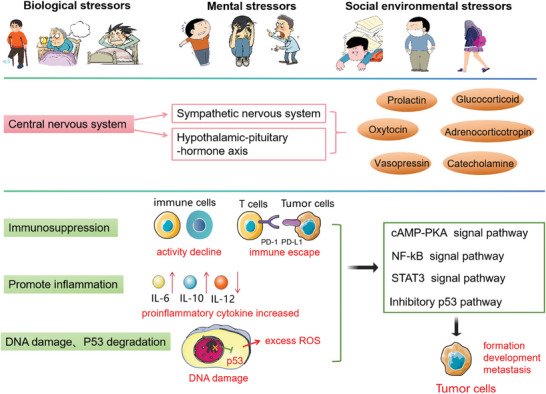
Psychological factors affect body function and the mechanism of inducing cancer.

Chronic stress produces stress hormones such as corticosteroids and catecholamines by activating the neuroendocrine system (HPA axis) and SNS. These stress hormones promote the occurrence and development of cancers through various mechanisms such as inducing DNA damage, increasing p53 degradation, and regulating the tumor microenvironment.^[^ ^113^ ^]^ Corticosteroids include glucocorticoids and corticosterones. Elevated glucocorticoid levels increase the activity of the negative regulator murine double minute 2 through induction of the serum‐and‐glucocorticoid‐regulated kinase and mediate the inhibition of p53^[^ ^114^ ^]^ (**Figure** **5**).

**Figure 6:**
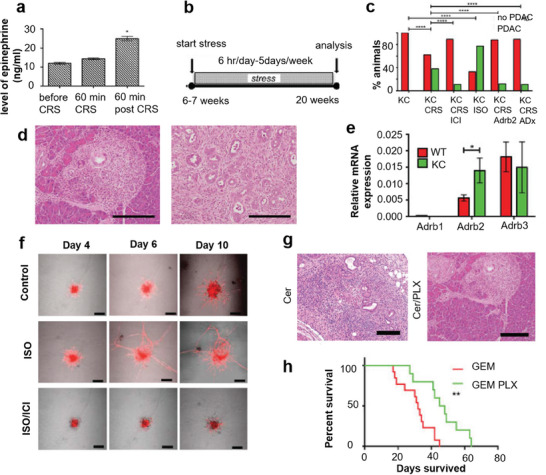
Chronic stress promotes tumor growth and metastasis. a) Systemic epinephrine levels in mice before, 60 min in stress, and 60 min after stress. b) Experimental set up: mice were stressed starting at 6 weeks of age for 6 h day^−1^, 5 days week^−1^ until 20 weeks of age. c) Comparison of incidence in different treatment groups. d) Representative images of pancreatic H&E slides from KC and KC CRS mice. e) Relative quantification of Adrb1, Adrb2, and Adrb3 mRNA expression in pancreata from WT mice and KC mice (*n* = 3, respectively). f) Confocal pictures of DRGs from E14.5 mTmG murine embryos cultured next to K8282 cells. g) Representative image of pancreatic H&E staining of cerulein‐injected KC mice at 20 weeks on control diet (Cer) and PLX‐7486 containing diet (Cer PLX); Pathological scoring of PanINs of pancreata depicted (*n* = 10 mice each group) at 20 weeks. h) Survival rate of tumor mice. Reproduced with permission.^[^ ^115^ ^]^ Copyright 2018, Cell Press.

Renz et al reported that the tumor‐promoting effect of catecholamines is mainly mediated by the β2 adrenergic receptor (ADRB2) activating the cyclic adenosine momophosphate‐protein kinase A signaling pathway, which is the main mechanism for enhancing tumor angiogenesis in vivo and promoting the growth of malignant cells.^[^ ^115^ ^]^ They found that chronic restraint stress (CRS) can lead to an increase in circulating systemic adrenaline levels (**Figure** **6**a), and they constructed a mouse model of prolonged stress (Figure 6b). Pathological scores at 20 weeks of age revealed the presence of pancreatic ductal adenocarcinoma (PDAC) lesions not observed in the unstressed mice, compared to 38% of the stressed mice (Figure 6c,d). ADRB2 is the main mediator of chronic stress‐induced cancer (including PDAC), and the expression of Adrb2 mRNA in the pancreas of Pdx1‐Cre (KC) is up‐regulated (Figure 6e). Further studies showed that catecholamines significantly increased neurite growth at days 4, 6, and 10, and pretreatment with ADRB2 antagonist therapy (ICI) almost completely blocked neurite growth (Figure 6f). These in vitro results suggest that ADRB2‐dependent nerve growth factor (NGF) upregulation contributes to increased axon interactions. Mice with tumors were injected with gemcitabine (GEM) or GEM and PLX (an inhibitor that blocks NGF secretion from ADRB2 for two weeks. Peripheral immunostaining showed a significant decrease in the PDAC nerve area in KC mice treated with GEM PLX, which is consistent with a decrease in NGF signaling (Figure 6g). Combined treatment with GEM PLX extended the survival of KPC mice from 32 days to 45 days (Figure 6h).

**Figure 7:**
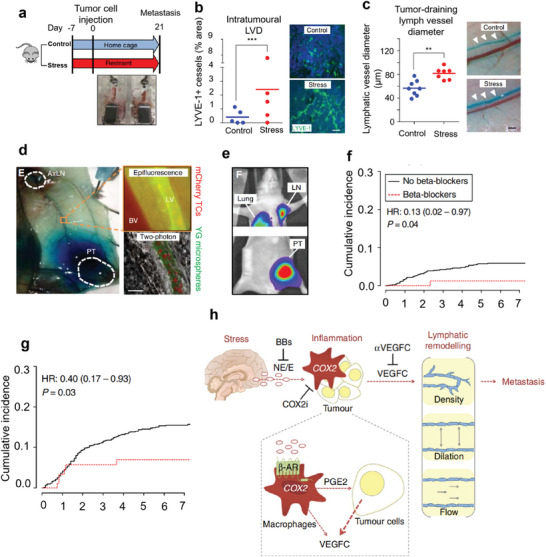
a) Stress model construction. b) Quantification and representative images of tumor LVD (LYVE‐1^+^, green; nuclear, blue) immunostaining of MDA‐MB‐231 orthotopic tumors. Scale bar, 200 mm (*n* = 5). c) Quantification and representative images of tumor‐draining lymphatic vessel diameter. d) The dye enters the axillary lymph nodes of the tumor, and the mcherry‐labeled MDA‐MB‐231 tumor cells spontaneously disperse from PT in situ. e) MDA‐MB‐231 breast cancer model showing PT, and spontaneous metastasis to draining lymph node (LN) and lung 21 days after tumor cell injection. BB use is associated with reduced lymph node f) and distant metastasis g). h) Stress‐induced lymphatic remodeling. Reproduced with permission.^[^ ^117^ ^]^ Copyright 2016, Springer Nature.

Chronic stress and stress hormones upregulate the expression of stress‐related pro‐inflammatory genes in circulating white blood cells, thereby increasing the release of pro‐inflammatory cells and the production of pro‐inflammatory cytokines, and can activate the aging‐inflammatory response without the trigger of an exogenous inflammation, thereby promoting tumorigenesis and metastasis.^[^ ^116^ ^]^ Le et al showed that chronic stress reorganizes the lymphatic network within and around tumors, providing pathways for tumor cells to escape.^[^ ^117^ ^]^ To study the effect of stress on lymphatic blood vessels in tumors, they built a stress model that used the expression of lymphatic vessel endothelial hyaluronan receptor 1 (LYVE‐1), a lymphatic endothelial cell marker in primary tumor sections, as a measure of tumor‐associated lymphatic vessel density (LVD) (**Figure** **7**a). Compared with control tumor‐bearing mice, chronic stress significantly increased tumor LYVE‐1 staining, suggesting that stress‐induced signaling can increase lymphatic networks within primary tumors (Figure 7b). Moreover, a comparison of lymphatic vessels between control and stressed mice showed that chronic stress caused significant lymphatic dilation of the primary tumor (Figure 7c). Dynamic observation of the lymph nodes showed that mCherry‐tumor cells transferred spontaneously from the primary tumor along the drainage lymphatics to the tumor drainage lymph nodes (Figure 7d). After 21 days of injection, mCherry‐tumor cells metastase to the tumor‐draining lymph nodes and lungs (Figure 7e). After the use of epinephrine blockers b‐adrenoceptor antagonist (BB), patients with triple‐negative breast cancer treated with BB showed a significant reduction in lymph node metastasis and distant metastasis (Figure 7f,g). They came up with that, stress hormones stimulate the pro‐tumorigenic immune cells to produce cytokines such as IL‐6 and IL‐10, and activate the cyclooxygenase‐2 (COX‐2)/prostaglandin E2 (PGE2) pathway to produce VEGF, which together affect the tumor microenvironment and inhibit tumor immunity (Figure 7h).

In addition, studies have shown that chronic stress leads to increased inflammation. Decreased IL‐12 and increased IL‐10 lead to selective T helper 1 (Th1) inhibition, thereby inhibiting cytotoxic T lymphocyte‐mediated cellular immunity and interferon production In addition, activated inflammatory cells can produce excess reactive oxygen species (ROS) to drive inflammation and mutation.^[^ ^118^ ^]^ The released cytokines can activate key transcription factors in precancerous cells such as nuclear factor‐κB (NF‐κB)^[^ ^119^ ^]^ and signal transducer and activator of transcription 3 (STAT3).^[^ ^120^ ^]^ Cytokines also attract more tumor‐promoting immune cells to maintain tumor‐associated inflammation by regulating the expression of many genes that inhibit tumor cell death, promote tumor cell survival, and induce chemokine production.

Substantial evidence suggests that depression is not only accompanied by inflammation but also by cell‐mediated immune activation and T‐cell and Th‐1‐like activations.^[^ ^121^ ^]^ A meta‐analysis showed a positive correlation between depression and inflammatory markers (C‐reactive protein (CRP), IL‐1, and IL‐6), and that there is a dose‐response relationship between depression and these inflammatory markers, lending strength to the contention that the cancer risk conferred by depression is not exclusive to patient populations. And there is a bidirectional relationship between depression and inflammation.^[^ ^122^ ^]^

Currently, optimal oncology care guidelines include screening and managing psychosocial issues.^[^ ^28^ ^]^ Antoni et al. believe that stress management interventions can be physiologically protective against tumor progression by enhancing protective immunity (e.g., immune surveillance), reducing chronic inflammatory processes, and suppressing immunosuppressive mechanisms.^[^ ^123^ ^]^ In breast cancer survivors, yoga and tai chi reduced pro‐inflammatory processes,^[^ ^124^ ^]^ and mindfulness‐based stress reduction increased the ratio of Th1/ Th2 cells,^[^ ^125^ ^]^ decreased NF‐κB activity, and increased anti‐inflammatory signaling and gene expression of type 1 interferons.^[^ ^126^ ^]^ In addition, psychosocial interventions enhanced protective immunity (such as increased gene expression of type 1 interferon, as well as serum levels of interferon‐gamma (IFN‐γ) and IL‐2, decreased Inflammatory processes (e.g., decreased expression of genes encoding IL‐1β, IL‐6, and tumor necrosis factor‐alpha (TNF‐α), increased prevalence of glucocorticoid receptor response elements).^[^ ^127^ ^]^

### 3.2 Nutrition

Many studies have explored and provided evidence of an association between diet and nutrition and cancer risk,^[^ ^128^ ^]^ adherence to diet guidelines for cancer prevention is consistently associated with an overall low incidence rate of cancer and mortality, including cancer at some specific sites.^[^ ^129^ ^]^ Diet and nutrition have been considered an effective cancer prevention strategy. Many diets and natural products have shown a potential role in preventing cancer.^[^ ^55^ ^]^ Such as polyphenols are naturally occurring micronutrients found in many food sources such as vegetables, red fruits, grains, spices, and green tea, etc. polyphenolic compounds such as flavonoids, curcumin, anthocyanins, resveratrol, ellagitannin, quercetin, catechin and so on have been reported to display many anticarcinogenic properties including their inhibitory effects on cancer cell proliferation, tumor growth, angiogenesis, metastasis, and inflammation as well as inducing apoptosis. In addition, they can modulate immune system response and protect normal cells against free radical damage.^[^ ^130^ ^]^

Isoflavones are mainly found in members of the Leguminosae family. Soy, lentils, beans, and chickpeas are sources of isoflavones; however, soybeans are legumes that contain abundant amounts of isoflavones.^[^ ^131^ ^]^ A meta‐analysis of prospective studies indicated that intake of isoflavones was significantly associated with reduced risk of lung and gastric cancer and nearly significant breast and colorectal cancers.^[^ ^82^ ^]^ Human and animal studies suggest that isoflavones exert their inhibitory effects on carcinogenesis and cancer progression by induction of apoptosis and inhibition of cell proliferation.^[^ ^132^ ^]^ The induction of apoptosis by isoflavone has been believed to be mediated through the regulation of multiple cell signaling pathways including NF‐κB,^[^ ^133^ ^]^ serine/threonine protein kinase B (Akt),^[^ ^134^ ^]^ mitogen‐activated protein kinases (MAPK),^[^ ^135^ ^]^ wingless (Wnt),^[^ ^136^ ^]^ Notch,^[^ ^137^ ^]^ p53,^[^ ^138^ ^]^ and androgen receptor (AR)^[^ ^139^ ^]^ signaling pathways.

Curcumin is a polyphenolic compound and secondary metabolite isolated from the rhizomes of turmeric (commonly known as turmeric) and is an herbaceous perennial plant in the ginger family. Curcumin has long been associated with health benefits and it has shown various therapeutic effects by regulating different molecular regulators and is used in traditional Indian medicine to treat various diseases.^[^ ^140^ ^]^ Curcumin has been shown to modulate multiple cell‐signaling pathways simultaneously, thereby mitigating or preventing many different types of cancers, including multiple myeloma and colorectal, pancreatic, breast, prostate, lung, head, and neck cancers, in both animal models and humans.^[^ ^141^ ^]^ Many studies have demonstrated that curcumin exhibits powerful anticancer, antioxidation stress, and anti‐inflammatory activities through regulatory signaling pathways, such as nuclear factor erythroid 2 related factor 2, NF‐κB, and epigenetics/epigenomics pathways of histones modifications, and DNA methylation. Therefore, curcumin has great potential to prevent colorectal cancer.^[^ ^142^ ^]^ Yang et al. found that curcumin can increase the expression of Bax in small‐cell lung cancer, decrease the expression of B‐cell lymphoma‐2 (Bcl‐2) and Bcl‐xL, thus inducing apoptosis and increase the level of intracellular ROS, and decrease mitochondrial membrane potential, induce the release of cytochrome c into cytoplasm, and then activate caspase‐9 and caspase‐3.^[^ ^143^ ^]^ In addition, curcumin can reduce the mitochondrial membrane potential and increase the apoptosis rate of gastric cancer cells, which is related to the impaired ATP‐sensitive potassium channel pathway.^[^ ^144^ ^]^

Anthocyanins are a class of water‐soluble flavonoids, widely existing in blueberries, eggplants, purple potatoes, black mulberries, black wolfberry and other edible plants. Wang et al. conducted a meta‐analysis of seven studies, which indicates a significant inverse association between total anthocyanin intake and colorectal cancer risk (R^2^ = 0.78; 95% CI, 0.64–0.95). Likewise, there was considerable evidence of a relationship between anthocyanin intake and colorectal cancer in the colon site (R^2 ^= 0.81; 95% CI, 0.71–0.92).^[^ ^62^ ^]^ It has been reported that the main potential molecular mechanism of the anti‐tumor effect of anthocyanins in vitro may be the inhibition of cancer cell growth by targeting receptor tyrosine kinases (RTKs) (e.g., epidermal growth factor receptor (EGFR), platelet‐derived growth factoe receptor (PDGFR), vascular endothelial growth factor (VEGF)/ vascular endothelial growth factor receptor (VEGFR) and acting on the Ras‐MAPK and phosphatidylin‐ositol‐3‐kinase (PI3K)/Akt cascade pathway. During cancer initiation, anthocyanins may prevent malignant transformation by targeting the MAPK pathway and AP‐1 factor and inhibiting RTK activity. Anthocyanins can initiate the expression of p21 and p27, whose products can combine with multiple cyclin‐dependent kinases (CDKs) to inhibit the expression of CDK‐1 and CDK‐2, further inhibiting the expression of cyclin‐B, cyclin‐A, and cyclin‐E, which promote the expression of CDK inhibitors and induce cancer cell arrest in G0/G1 and G2/M phases. During tumor development, anthocyanins can induce cancer cell apoptosis by activating ROS and JNK/p38‐MAPK‐mediated caspases. Anthocyanins may also exert their anti‐metastatic activity by targeting the VEGF signaling pathway and extracellular matrix degradation (via matrix metalloproteinase (MMP)‐2, MMP9) In addition, the abnormal overexpression and secretion of inflammatory factors are the key to the occurrence of tumors, so chronic inflammation is usually the harbinger of tumor. Anthocyanins may also inhibit inflammation through the PI3K/Akt and NF‐κB pathways, inhibit the expression of COX‐2 and iNOS, regulate the expression of phase II antioxidant enzymes through the Nrf2/ARE signaling system, and achieve anti‐oxidation, thereby preventing cancer.^[^ ^145^ ^]^

Resveratrol is a natural polyphenolic compound found in many plants, including grapes (especially skins), blueberries, peanuts, and red wine.^[^ ^146^ ^]^ Resveratrol can induce apoptosis of cancer cells through the p53‐dependent activation of pro‐apoptotic proteins, such as Bax, NOXA, BUMA, etc. Also, resveratrol induces cancer cell death by autophagy through upregulation of Sirt1 and AMPK. Resveratrol inhibited metastasis by inhibiting epithelial‐to‐mesenchymal transition (EMT) through down‐regulating TGF‐β1/Smads, Wnt/β‐catenin, PI3K/Akt/NF‐κB and Gli1 signaling pathways. Resveratrol inhibits angiogenesis through HIF‐1α‐dependent inhibition of VEGF.^[^ ^147^ ^]^

Ellagitannin is the main component of the high content of polyphenols in pomegranate (Punica granatum L.) known as “nature's power fruit”, which has potent antioxidant and anti‐inflammatory properties.^[^ ^55^ ^]^ Pomegranate extract (ellagitannin) can inhibit the growth of MCF‐7 breast cancer cells by inducing cell cycle arrest in the G2/M phase, which may be related to down‐regulating homologous recombination and making cancer cells sensitive to double‐strand breaks.^[^ ^148^ ^]^ Ellagitannins, including punicalagin, are broken down after intestinal pH and/or gut microbiota are exposed to ellagic acid. Ellagitannins are further metabolized by intestinal microbiota into various urolithins, including the most biologically relevant, urolithin A.^[^ ^149^ ^]^ Punicalagin has been shown to increase the growth inhibition and apoptosis of two human PCa cell lines (PC3 and LNCaP), suggesting that the bioactive substances in pomegranate have potential anticancer activity.^[^ ^150^ ^]^ Urolithin A has been shown to repress three PCa cell lines with differing p53 genotypes (LNCaP (*p*53 +/+), 22RV1 (*p*53‐/+), and PC3 (*p*53‐/‐), including induction of apoptosis.^[^ ^151^ ^]^

Quercetin (3,3′,4′,5,7‐pentahydroxylflavone) is a naturally occurring polyphenolic flavonoid that is widely found in different fruits and vegetables, such as apples, oranges, grapes, blueberries, onions, dill, and beans.^[^ ^152^ ^]^ Quercetin has been reported to protect against various cancers, such as lung, prostate, liver, breast, colon, and cervical cancers.^[^ ^153^ ^]^ Lam et al. found that frequent intake of quercetin‐rich foods was inversely associated with lung cancer risk (OR = 0.49; 95% CI: 0.37–0.67; *p* < 0.001).^[^ ^154^ ^]^ Quercetin has been shown to modulate several signal transduction pathways involving MEK/ERK and Nrf2/keap1, which are associated with the processes of inflammation and carcinogenesis.^[^ ^155^ ^]^ Nguyen et al. showed that quercetin not only increases apoptosis but also inhibits cell cycle progression; moreover, it can promote FasL mRNA expression and enhance the activity of p51, p21, and GADD45 signaling pathways.^[^ ^156^ ^]^ Seo et al. found that quercetin induces caspase‐dependent extrinsic apoptosis upregulating the levels of cleaved caspase‐8 and cleaved caspase‐3, and inducing the cleavage of poly (ADP‐ribose) polymerase (PARP).^[^ ^157^ ^]^ In addition, quercetin induced cell apoptosis in a mitochondrial‐dependent manner, as shown by the reduction in mitochondrial membrane potential, the activation of caspase‐3 and −9, and the down‐regulation of Bcl‐2, as well as the upregulation of Bax and cytochrome c.^[^ ^158^ ^]^

Catechins are found in green tea, many types of fruit, red wine, and chocolate. Green tea, a rich source, contains up to 200 mg of catechins in a cup of tea.^[^ ^159^ ^]^ It is generally agreed that green tea's cancer chemoprophylaxis effects are mediated by its polyphenols known as catechins. The major catechins in green tea are (‐)‐epigallocatechin‐3‐gallate (EGCG), (‐)‐epicatechin‐3‐gallate, (‐)‐epigallocatechin and (‐)‐epicatechin. EGCG is the major catechin in green tea and accounts for 50%–80% representing 200–300 mg/brewed cup of green tea.^[^ ^160^ ^]^ A cohort study demonstrated that the increased tea intake was associated with lower hepatocellular cancer risk (HR, 0.41; 95% CI, 0.22–0.78), and nine prospective cohort studies of China, Japan, and Singapore observed that the consumption of green tea was effective in reducing the risk of liver cancer in women (RR, 0.78; 95% CI, 0.64–0.96).^[^ ^161^ ^]^ Additionally, a cohort study of Chinese adult men found that green tea drinkers had significantly lower cancer overall mortality (<5 g day^−1^: HR, 0.86; 95% CI, 0.78‐0.98; 5–10 g day^−1^: HR, 0.92; 95% CI, 0.83‐1.00; >10 g day^−1^, HR, 0.79; 95% CI, 0.71 = 0.88).^[^ ^162^ ^]^ Many of the cancer chemoprophylaxis properties of green tea are mediated by EGCG that induce apoptosis and promote cell growth arrest by altering the expression of cell cycle regulatory proteins, activating killer caspases, and suppressing oncogenic transcription factors and pluripotency maintain factors. In vitro, studies have demonstrated that EGCG blocks carcinogenesis by affecting a wide array of signal transduction pathways including JAK/STAT, MAPK, PI3K/AKT, Wnt, and Notch.^[^ ^163^ ^]^ In addition, catechin extract could induce apoptosis of PC‐3 cells through decreased Bcl‐2 expression and increased cytochrome c expression to activate caspase‐3, caspase‐8, and caspase‐9.^[^ ^164^ ^]^ Tumorigenesis is a multistep process that can be activated by any of various environmental carcinogens (such as cigarette smoke, industrial emissions, gasoline vapors), tumor promoters (such as phorbol esters and okadaic acid), and inflammatory agents (such as TNF‐α and H_2_O_2_). These cancer‐promoting agents are known to modulate the transcription machinery factors (e.g., NF‐κB, AP‐1, STAT3), antiapoptotic proteins (e.g., Akt, Bcl‐2, Bclxl), proapoptotic proteins (e.g., caspases, PARP), protein kinases (e.g., IKK, JNK, MAP kinase), cell cycle proteins (e.g., cyclins, CKDs), cell adhesion molecules, COX−2, and growth factor signaling pathways. (Green tea catechin, EGCG): Mechanisms, perspectives and clinical applications, Biochemical Pharmacology, 2011)

Glucosinolates are sulfur‐rich, anionic natural products that are present in certain cruciferous vegetables (cabbage, cauliflower, broccoli) and condiments (mustard, horseradish, wasabi), it is called the myrosinase sulfur endogenous glycosidase enzymes hydrolysis to produce several different products (e.g., isothiocyanates, thiocyanates, and indoles).^[^ ^165^ ^]^ Liu et al. conducted a meta‐analysis of 13 epidemiological studies (11 case‐control studies and 2 cohort studies), the combined results of all studies showed that high cruciferous vegetable intake was significantly associated with reduced breast cancer risk (R^2 ^= 0.85, 95% CI, 0.77–0.94).^[^ ^51^ ^]^ Xu et al. demonstrated that cell apoptosis depends on the caspase pathway during isothiocyanate‐induced human leukemia, and the JNK pathway may play a supporting role.^[^ ^156^ ^]^ A study found that sulforaphane (a type of isothiocyanate) induced apoptosis in prostate cancer cells by activating caspases ERK1/2 and Akt, increasing p53 and Bax protein levels.^[^ ^166^ ^]^ A study found that sulforaphane (a type of isothiocyanate) induced apoptosis in prostate cancer cells by activating caspases, ERK1/2 and Akt, increasing p53 and Bax protein levels.^[^ ^167^ ^]^ Satyan et al. demonstrated that isothiocyanate exhibits cytotoxicity towards OVCAR‐3 cells and induces apoptosis via caspase‐9 and −3 pathways, and inhibits Akt, ERK1/2 survival signaling, and c‐Myc while activating pro‐apoptotic p38 and JNK1/2.^[^ ^168^ ^]^ Trachootham et al found that exposure of chronic lymphocytic leukemia cells to β‐phenylethyl isothiocyanate (PEITC) induced severe glutathione depletion, ROS accumulation, and oxidation of mitochondrial cardiolipin leading to massive cell death.^[^ ^169^ ^]^ In addition, isothiocyanates may induce cell cycle arrest in different phases in a cell line‐dependent manner. Allyl isothiocyanate arrested HL‐60 cells at the G1 phase, whereas benzyl isothiocyanate arrested the cells at both the G1 and the G2/M phases.^[^ ^170^ ^]^ Indole‐3‐methanol, which is an indole, can selectively induce G1/S arrest of the cell cycle and apoptosis of tumor cells, which is considered to be a key process for preventing tumor growth.^[^ ^171^ ^]^ These studies demonstrate that they exhibit antitumor activity by affecting multiple pathways including apoptosis, MAPK signaling, oxidative stress, and cell cycle progression.

There are three provitamin A carotenoids (β‐carotene, α‐carotene, and β‐cryptoxanthin) and three non‐provitamin A carotenoids (lycopene, lutein, and zeaxanthin) that can be found routinely in human plasma and tissues. These carotenoids have been studied for their potential beneficial roles against cancer development (e.g., lung, liver, prostate, breast, colorectal, and stomach). Some research that the biological functions of β‐cryptoxanthin and lycopene are mediated, partially via their oxidative metabolites, through their effects on key molecular targeting events, such as NF‐κB signaling pathway, RAR/PPARs signaling, SIRT1 signaling pathway, and p53 tumor suppressor pathways.^[^ ^172^ ^]^ Some results suggest that lycopene may inhibit the carcinogenic process through the inactivation of the growth factor (PDGF, VEGF, and IGF) induced PI3K/AKT/PKB and Ras/RAF/MAPK signaling pathways. When active, these pathways activate transcription factors (NF‐κB, AP‐1, and SP‐1) that regulate the expression of genes that control cellular processes such as proliferation, cell cycle, apoptosis, inflammation, angiogenesis, invasion, and metastasis. In addition, some results suggest that lycopene blocks cell‐cycle progression from G1 to S phase, predominantly by reducing the levels of cyc D and E and subsequently by inactivating CDK2 and 4 and decreasing the hyperphosphorylation of Rb. Furthermore, lycopene increases the expression of CDK inhibitors including p21 and p27, as well as the tumor suppressor gene p53, and decreases the expression of Skp2.^[^ ^173^ ^]^

Capsaicin is a bioactive phytochemical abundant in red and chili pepper, which has been shown to alter the expression of several genes involved in cancer cell survival, growth arrest, angiogenesis, and metastasis.^[^ ^174^ ^]^ Lau et al. found that capsaicin can promote apoptosis by downregulating the transient receptor potential vanilloid (TRPV) receptor in human small‐cell lung cancer cells.^[^ ^175^ ^]^ Besides, capsaicin can inhibit the proliferation of human gastric cancer cells (AGS cells) and induce apoptosis by increasing cleaved caspase‐3, reducing Bcl‐2, and reducing the expression of phosphorylated ERK 1/2, p38 MAPK, or JNK.^[^ ^176^ ^]^ In addition, Wutka et al. showed that capsaicin has an effective inhibitory effect on the proliferation, migration, and invasion of human cholangiocarcinoma cells, which was associated with regulating the Hedgehog signaling pathway.^[^ ^177^ ^]^ We summarized the associations and mechanisms between the above nutrients and cancer in **Table** **3**.

**Table 3:** Nutrients and active components affect the relationship and mechanism of cancer occurrence and development.

Source objects	Active ingredient	Relationship with tumor	Mechanism
Soybeans	Isoflavones	Intake of isoflavones was significantly associated with a reduced risk of lung, stomach, breast and colorectal cancer	Isoflavones induce apoptosis and inhibit cell proliferation by regulating signaling pathways including NF‐κB, Akt, MAPK, Wnt, Notch, p53, and AR to inhibit carcinogenesis and cancer progression.^[^ ^133^, ^134^, ^135^, ^136^, ^137^, ^138^, ^139^ ^]^
Ginger	Curcumin	Curcumin has been shown to regulate multiple cell signaling pathways simultaneously to mitigate or prevent many different types of cancer, including multiple myeloma and colorectal, pancreatic, breast, prostate, lung, head and neck cancers.	Curcumin exhibits anti‐oxidative stress and anti‐inflammatory activity through NF‐κB, histone modification, and DNA methylation.^[^ ^140^ ^]^ Curcumin induces the increase of ROS level, decreases mitochondrial membrane potential, and activates caspase‐9 and caspase‐3 to induce apoptosis of tumor cells.^[^ ^142^ ^]^ Curcumin can increase the apoptosis rate of gastric cancer cells by damaging the ATP‐sensitive potassium channel pathway.^[^ ^144^ ^]^
Blueberry, eggplant, purple potato, black mulberries, black wolfberry	Anthocyanins	Anthocyanins prevent cancer through anti‐inflammatory and antioxidant activities; Anthocyanin intake was negatively correlated with colorectal cancer.^[^ ^62^ ^]^	Anthocyanidins inhibit cancer cell growth by targeting RTKs (EGFR, PDGFR, VEGF/VEGFR) and acting as Ras‐MAPK and PI3K/Akt level pathways. Anthocyanins induce apoptosis of cancer cells by activating ROS and JNK/ p38‐mapk mediated caspase. Anthocyanins may also inhibit inflammation through PI3K/Akt and NF‐κB pathways, inhibit the expression of COX‐2 and iNOS, and regulate the expression of phase II antioxidant enzymes through Nrf2/ARE signaling system to achieve the purpose of antioxidant.^[^ ^145^ ^]^
Grapes (especially the skins), blueberries, peanuts, and red wine	Resveratrol	Resveratrol inhibited tumor metastasis, inhibited tumor angiogenesis, and induced tumor apoptosis.^[^ ^146^ ^]^	Resveratrol can activate pro‐apoptotic proteins such as Bax, NOXA, and BUMA in a p53‐dependent manner. Autophagy death was induced by up‐regulation of Sirt1 and AMPK. Down‐regulating TGF‐β1/Smads, Wnt/β‐catenin, PI3K/Akt/NF‐κB, and Gli1 signaling pathways inhibited EMT. Angiogenesis is inhibited by HIF‐1α‐dependent inhibition of VEGF135.^[^ ^147^ ^]^
Pomegranate	Ellagitannin	Ellagitannin has powerful anti‐inflammatory and antioxidant functions and has an inhibitory effect on breast cancer and prostate cancer.^[^ ^55^ ^]^	Inhibit the growth of MCF‐7 breast cancer cells by inducing cell cycle arrest in the G2/M phase.^[^ ^148^ ^]^
Apples, oranges, grape, blueberry, onions, dill and beans	Quercetin	Quercetin has been reported to protect against a variety of cancers, such as lung, prostate, liver, breast, colon, and cervical cancers.^[^ ^152^ ^]^	Quercetin regulates the inflammatory and carcinogenic pathways MEK/ERK, Nrf2/keap1; In addition, it can promote the expression of FasL mRNA and enhance the activity of p51, p21, and GADD45 signaling pathways. Quercetin‐induced apoptosis by up‐regulating the levels of lytic caspase‐8 and lytic caspase‐3.^[^ ^154^, ^155^, ^156^, ^157^, ^158^ ^]^
Green tea	Catechins	Green tea intake significantly reduced the risk of liver cancer; Green tea drinkers had a significant reduction in overall cancer mortality.^[^ ^159^ ^]^	Many of the cancer chemoprophylaxis properties of green tea are mediated by EGCG, which blocks carcinogenesis by affecting a range of signal transduction pathways including JAK/STAT, MAPK, PI3K/AKT, Wnt, and Notch.^[^ ^163^, ^164^ ^]^
Cabbage, cauliflower, broccoli, mustard, horseradish, wasabi	Glucosinolates	High intake of cruciferous vegetables was significantly associated with a reduced risk of breast cancer.^[^ ^165^ ^]^	Sulforaphane induces apoptosis of prostate cancer cells by activating caspase, ERK1/2, and Akt, increasing p53 and Bax protein levels. Exposure of chronic lymphocytic leukemia cells to PEITC results in severe glutathione depletion, ROS accumulation, and mitochondrial cardiolipin oxidation, leading to massive cell death. In addition, isothiocyanates induced cell cycle arrest and showed antitumor activity.^[^ ^167^, ^168^, ^169^, ^170^, ^171^ ^]^
Carrot	Carotene	Carotene has been investigated for its potential anti‐cancer effects in lung, liver, prostate, breast, colon, and stomach cancer.	β‐cryptoxanthin and lycopene regulate the NF‐κb, RAR/PPARs, SIRT1, and p53 tumor suppressor pathway. These transcription factors regulate the expression of genes that control cellular processes such as proliferation, cell cycle, apoptosis, inflammation, angiogenesis, invasion, and metastasis, thus achieving anti‐tumor effects.^[^ ^172^, ^173^ ^]^
Peppers	Capsaicin	It has been shown to alter the expression of several genes involved in cancer cell survival, growth arrest, angiogenesis, and metastasis.^[^ ^174^ ^]^	Capsaicin promotes apoptosis by down‐regulating TRPV receptors in human small‐cell lung cancer cells. In addition, capsaicin inhibited proliferation and induced apoptosis of human gastric cancer cells by increasing cleavage of Caspase‐3, decreasing expression of Bcl‐2, and decreasing phosphorylated ERK 1/2, p38 MAPK, or JNK166. Capsaicin regulates the Hedgehog signaling pathway and has an effective inhibitory effect on the proliferation, migration, and invasion of human bile duct cancer cells.^[^ ^175^, ^176^, ^177^ ^]^

### 3.3 Exercise

**Figure 8:**
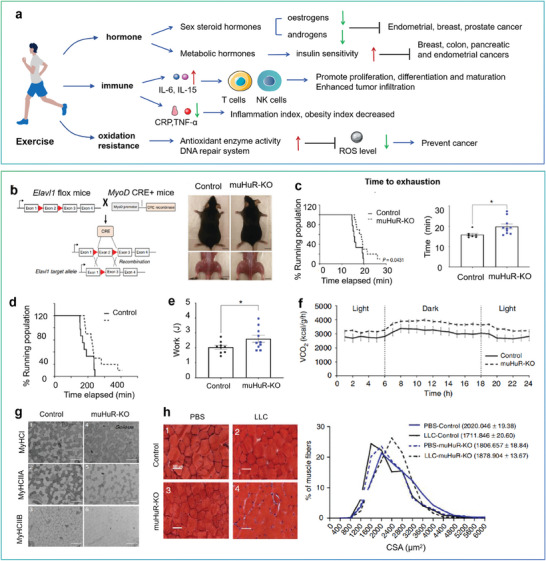
The effects of exercise on cancer. a) The mechanism of exercise‐induced changes in the body and the occurrence of cancer. b) Diagram depicting the tissue‐specific knockout strategy. c–e) Physical performance was evaluated in age‐matched control and muHuR‐KO mice by performing a treadmill exhaustion test. Three parameters were measured with this test: c. Time to exhaustion (left panel; survival plot showing the percentage of mice running at indicated time points. Right panel; mean duration of the run). d. Running distance (left panel; survival plot showing the percentage of mice running at indicated distances. Right panel; mean distance ran) and e. Work performed during the test. f) The amount of VO_2_ and VCO_2_. g) Representative photomicrographs of serial sections of soleus muscles from control and muHuR‐KO mice taken after immunostaining with anti‐Myosin Heavy Chain (MyHC) antibodies type I, type IIA and type IIB. h) Left panel: representative photomicrographs of gastrocnemius muscle sections from control and muHuR‐KO mice taken after H&E staining. Scale bars = 100 µm. Right panel: frequency histogram showing the distribution of muscle fiber CSA in the gastrocnemius muscles from control and muHuR‐KO mice bearing or not LLC tumors (*n* = 4 mice per group). A total of 500 fibers per muscle were used for the CSA analysis.^[^ ^187^ ^]^ Copyright 2019, Springer Nature.

Physical activity can increase insulin sensitivity, reduce the concentration of hormones in plasma, increase the amount of glucose entering muscles, and reduce the synthesis of fatty acids. Insulin resistance and hyperinsulinism are closely related to obesity, intra‐abdominal fat, circulating adipokines, and inflammatory factors. These components regulate the development of cancer (**Figure** **8**a).^[^ ^178^ ^]^

In 2008, McTiernan published a seminal paper showing that physical activity was associated with cancer prevention through exercise‐ dependent reductions in cancer risk factors, such as sex hormones, insulin/IGF, and inflammatory markers.^[^ ^179^ ^]^ Exercise has been widely shown to regulate the cellular immune system because cytotoxic immune cells are mobilized into circulation during exercise by mechanisms involved in blood flow‐induced shear stress and adrenergic signaling.^[^ ^180^ ^]^ During exercise, physical activity modulates inflammatory status and cytokine levels (e.g., IL‐6, IL‐10, and IL‐1Rα).^[^ ^181^ ^]^ In a more indirect manner, exercise‐induced actin may affect the activity of immune cells through the release of immune regulatory cytokines such as IL‐6, IL‐7, and IL‐15.^[^ ^182^ ^]^ Many studies have confirmed that IL‐15 plays an important role in promoting NK and T cell proliferation, differentiation, and maturation.^[^ ^183^ ^]^ Pedersen et al. demonstrated that the presence of IL‐6 in circulation is most pronounced during muscle movement, and its appearance precedes that of other cytokines. Then came the IL‐1Rα and the anti‐inflammatory factor IL‐10. Chemokine, IL‐8, and macrophage inflammatory protein were elevated after vigorous exercise. In most exercise studies, TNF‐α does not change. In humans, IL‐6 increases exponentially with exercise, and the magnitude of IL‐6 increase depends on exercise duration, intensity, and muscle mass. After exercise, the basal plasma IL‐6 concentration can increase by 100 times. They tested 73 different types of exercise, and although the degree of increase was different, the trend of IL‐6 increase was the same.^[^ ^184^ ^]^ In addition, the findings suggest that long‐term exercise training reduces systemic levels of CRP, TNF‐α, IL‐6, and other pro‐inflammatory factors. Systemic anticancer response induced by endurance exercise training. Production of large but transient myosin, catecholamine during each acute exercise; baseline levels of cancer risk factors decreased modestly over time. They propose that acute systemic responses to exercise factors, such as increased catecholamines and muscle factors, drive the direct anti‐cancer effects of exercise mediated through their cumulative effects.^[^ ^185^ ^]^ IL‐6, TNF‐α, leptin, and CRP are recognized markers of inflammation. These markers are strongly positively correlated with obesity and negatively correlated with physical activity.^[^ ^186^ ^]^

Recently, Sanchez et al have revealed the mechanism by which skeletal muscle enhances exercise endurance and prevents muscle atrophy caused by cancer. They first created an Elavl1 muscle‐specific knockout mouse (muHuR‐KO) to study the in vivo role of HuR in muscle formation and muscle physiology (Figure 8b). Treadmill fatigue tests showed that the fatigue time and running distance of the muHuR‐KO mice were significantly longer than those of the control group (Figure 8c,d). In this test, the muHuR‐KO mice completed 20% more work than the control mice (Figure 8e), indicating increased exercise endurance in the muHuR‐KO mice. Increased endurance is often associated with an increase in the oxidative capacity of skeletal muscle fibers. They monitored the rate of oxygen consumption and carbon dioxide production during the animals' movements. The results showed that the Oxygen consumption (VO_2_) consumption rate and carbon dioxide production (VCO_2_) rate of muHuR‐KO mice were higher than those of the control group (Figure 8f). Specific disruption of the HuR gene in muscle improves exercise endurance and oxygen consumption. In general, increased endurance is associated with a significant increase in the proportion of type I fibers. Compared with the control animals, the enrichment of type I fibers in the soleus muscle of muHuR‐ko mice was ≈17%, while the enrichment of type IIA fibers was ≈16% (Figure 8g). Some reports suggest that in advanced stages, many cancers preferentially target type II glycolytic fibers, triggering rapid muscle atrophy, a fatal disease also known as cachexia‐induced muscle atrophy. Lewis lung cancer (LLC) cells, a cancer cell model widely used to trigger muscle atrophy in C57BL/6 mice. Cross‐sectional area analysis of muscle fibers in LCC‐control mice was reduced compared to LCC‐muHuR‐KO mice (Figure 8h). These results suggest that specific loss of HuR in skeletal muscle protects mice from cancer‐induced muscle atrophy.^[^ ^187^ ^]^

Exercise can reduce oxidative damage by increasing a variety of antioxidant enzymes, enhancing the DNA repair system, and improving the intracellular protein repair system. In addition to altering the processes related to tumorigenesis, exercise may also play a role in preventing cancer by inhibiting the carcinogenic processes including the scavenging of reactive ROS and the progression stage. Studies examining the relationship between exercise and chronic inflammation indicate that exercise may reduce proinflammatory mediators and reduce the state of low‐level chronic inflammation. Additionally, exercise has been shown to enhance the components of the innate immune response (i.e., macrophage and NK cell function).^[^ ^188^ ^]^

### 3.4 Smoking

**Figure 9:**
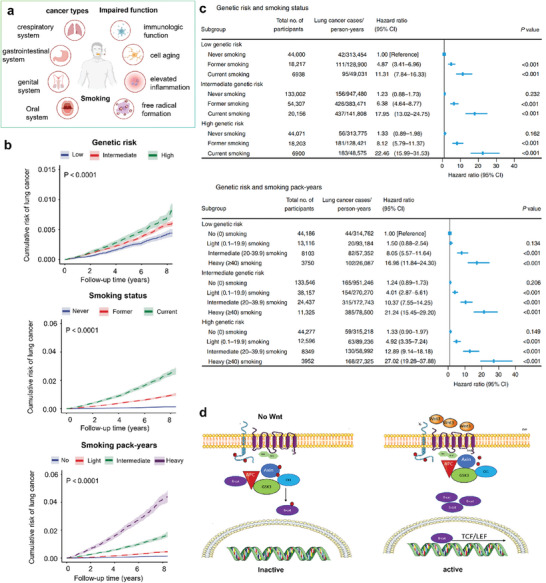
Smoking is highly associated with the risk of many types of cancer. a) Smoking causes many kinds of cancer and damage to the body. b) Cumulative risk of incident lung cancer according to genetic risk or smoking. c) Risk of incident lung cancer according to a combination of genetic risk and smoking.^[^ ^192^ ^]^ Copyright 2022, Springer Nature. d) Wnt/β‐catenin pathway in the presence and absence of Wnt signal.^[^ ^196^ ^]^ Copyright 2023, Elsevier Inc.

Tobacco is one of the leading causes of cancer worldwide, which can be attributed to the development of ≈20 types of malignant tumors, and is primarily responsible for >70% of lung cancer cases worldwide.^[^ ^189^ ^]^ Tobacco smoke is a complex chemical mixture containing at least 60 carcinogens. Many of these are believed to contribute to the development of cancer by causing DNA damage. If these errors are replicated, they can lead to an accumulation of genetic mutations, which in turn increases the likelihood of cancer‐causing genes acquiring additional mutations.^[^ ^190^ ^]^ In addition, cigarette smoke causes diverse changes in the immune system, resulting in heightened constitutive inflammation, skewing of adaptive T‐cell‐mediated immune, impaired response to pathogens, and suppression of anti‐tumor immune cell function. The most harmful substances in cigarettes are nicotine, acrolein, benzopyrene, cadmium, etc., which can induce inflammation and carcinogenesis by interacting with immune cells, regulating inflammatory mediators, and reshaping the tumor microenvironment. The molecular mechanisms by which smoking causes inflammation and cancer include the induction of DNA damage, epigenetic or chromatin modification, and activation of carcinogenic signaling pathways (**Figure** **9**a).^[^ ^191^ ^]^

To date, there is a great correlation between smoking and lung cancer risk, which is beyond doubt. Zhang et al conducted a prospective cohort study on the association of smoking and polygenic risk with lung cancer incidence.^[^ ^192^ ^]^ During a multi‐year follow‐up of 2454915 people (median follow‐up [interquartile interval] was 7.2 [6.5–7.8] years), 1687 cases of lung cancer developed. The participants who developed lung cancer were slightly older, more male, smoked more, were less physically active, and had an unhealthy diet. They found that the incidence and HR of lung cancer increased with the change in smoking status and the increase in smoking age (Figure 9b). In each genetic risk group, the incidence and HR of lung cancer increased with worsening smoking status and increasing pack years. There was no significant difference in the risk of lung cancer in the high genetic risk but never smoking group compared to the low genetic risk and never smoking groups. The HR in the low genetic risk but current smoking group was 11.31 (95% CI, 7.84 to 16.33). The risk was highest in those with high genetic risk and smokers compared to those with low genetic risk and never smokers (HR, 22.46 [95% CI, 15.99–31.53]). Individuals with high genetic risk and heavy smoking are at much higher risk (Figure 9c). Cook et al. conducted a pooled analysis that smoking cessation appears to reduce the risk of esophageal adenocarcinoma (EAC), and compared with current smokers, people who quit smoking for 10 years or more have a ≈30% reduced risk of EAC.^[^ ^193^ ^]^ Aune et al. conducted a meta‐analysis of prospective studies and found that current smoking was associated with 49%, 93%, and 62% increases in the relative risk of acute pancreatitis, chronic pancreatitis, and acute/ chronic pancreatitis combined compared to never‐smokers, respectively, while the corresponding results for former smokers showed 24%, 30% and 29% increases in the relative risk compared to never smokers. In a dose‐response analysis, smoking 10 cigarettes per day increased the risk of acute pancreatitis and acute/chronic pancreatitis combined by 30% and 28%, respectively, and the risk of combined acute, chronic or acute and chronic pancreatitis increased by 10%–22% per smoking 10 pack years.^[^ ^194^ ^]^ Another comprehensive review and meta‐analysis confirmed that smoking increases the risk of kidney cancer by ≈40%, and the risk continues to increase with the intensity and duration of smoking.^[^ ^195^ ^]^

Recently, Malyla et al. revealed that smoking induces lung cancer tumorigenesis by upregulating the WNT/ beta‐catenin signaling pathway.^[^ ^196^ ^]^ They co‐cultured cigarette smoke extract (CSE) with healthy human bronchial epithelial cells (16HBE14o) and detected elevated levels of WNT/β‐catenin RNA, including WNT3, DLV3, Axin1 and β‐catenin. Furthermore, this co‐culture resulted in an enhanced migratory capacity of the cells CSE‐treated 16HBE14o cells to induce tumorigenic protein and gene expression in healthy bronchial epithelial cells. In the absence of WNT signaling, β‐catenin is phosphorylated by the Axin complex, leading to β‐catenin degradation. In the presence of WNT signaling, the Axin complex is located in the cell membrane and prevents the degradation of β‐catenin, leading to various downstream effects (Figure 9d).

### 3.5 Drinking Alcohol

**Figure 10:**
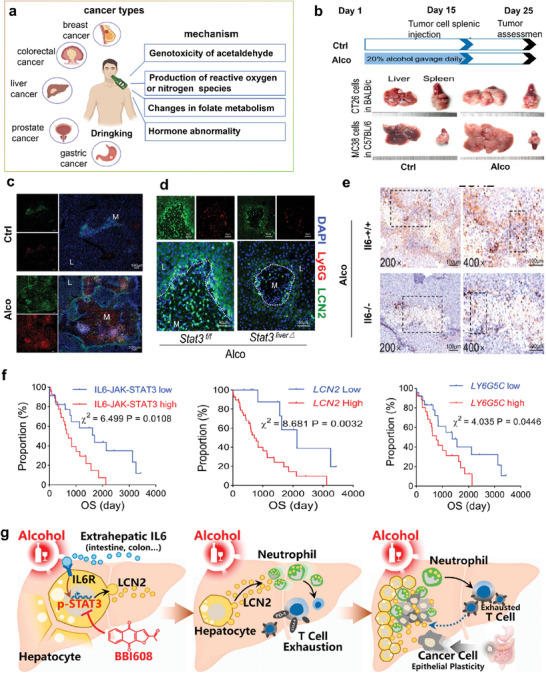
Alcohol‐related cancer risk. a) Alcohol intake induces multiple cancers and their mechanisms. b) Alcohol pretreatment promoted colon cancer cell metastasis to the liver in BALB/c (Ctrl, *n* = 8; Alco, *n* = 7) and C57BL/6 (*n* = 5) mice. c) Immunofluorescence of C57BL/6 mouse liver tissue sections under low‐power field. Alcohol promoted liver LCN2 expression and neutrophil (labeled by Ly6G) infiltration (*n* = 5). Alcohol‐induced neutrophil infiltration was reversed by knockout of d) hepatocyte Stat3 (*n* = 5) and e) systemic IL‐6 (n = 5), respectively. “L” and “M” denote liver and metastases, respectively. IOD, integral optical density. ^*^ *p* < 0.05, ^**^ *p* < 0.01, ^***^ *p* < 0.001. f) Kaplan‐Meier overall survival analysis by the IL‐6‐JAK‐STAT3 signature and LCN2 and LY6GC count values. g) Alcohol reshapes the liver premetastatic niche mechanism map of cancer through extrahepatic and intrahepatic crosstalk‐mediated immune escape.^[^ ^203^ ^]^ Copyright 2023, Cell Press.

According to the WHO, alcoholism is the leading cause of more than 200 diseases and injuries and is related to premature death and disability. In 1988, the IARC categorized alcohol as a Group 1 human carcinogen.^[^ ^197^ ^]^ In addition, the IARC believes that drinking alcohol is a cause of breast, colorectal, laryngeal, liver, esophageal, oral, and pharyngeal cancers.^[^ ^198^ ^]^ The biological mechanisms of alcohol‐induced cancer are not fully understood but may include the genotoxic effects of acetaldehyde, the production of reactive oxygen or nitrogen species, changes in folate metabolism, increased estrogen concentration, or as a solvent for tobacco metabolites (**Figure** **10**a).^[^ ^199^ ^]^

Many epidemiological studies explore the relationship between human drinking and cancer risk. A meta‐analysis of the incidence of alcohol‐related liver cancer found that there was a significant correlation between the risk of liver cancer and high‐level alcohol intake. The dose‐risk curve suggested a linear relationship with increasing alcohol intake in drinkers, with an estimated excess risk of 46% for 50 g of ethanol per day and 66% for 100 g per day.^[^ ^200^ ^]^ Bagnardi et al. conducted a comprehensive dose‐response meta‐analysis of alcohol consumption and site‐specific cancer risk. Relative risks for heavy drinkers compared with nondrinkers and occasional drinkers were 5.13 for oral and pharyngeal cancer, 4.95 for oesophageal squamous cell carcinoma, 1.44 for colorectal, 2.65 for laryngeal and 1.61 for breast cancer; for those neoplasms, there was a clear dose risk relationship. Heavy drinkers also had a significantly higher risk of cancer of the stomach (R^2 ^= 1.21), liver (2.07), gallbladder (2.64), pancreas (1.19) and lung (1.15). There was an indication of a positive association between alcohol consumption and the risk of melanoma and prostate cancer.^[^ ^201^ ^]^ In another dose‐response analysis, there was a statistically significant linear trend with breast cancer risk increasing gradually by total alcohol and wine doses: when 10 g was added daily, the risk of breast cancer increased by 10.5% (R^2^ = 1.10, 95% CI, 1.08–1.13 total alcohol) and 8.9% in wine (R^2^ = 1.08, 95% CI, 1.04–1.14), and for postmenopausal women, for every 10 g of total alcohol intake, the risk will increase by 11.1% (R^2^ = 1.11, 95% CI, 1.09–1.13).^[^ ^202^ ^]^

More recently, Qiu et al. revealed that alcohol reshapes the liver pre‐metastatic niche of cancer through extrahepatic and intrahepatic crosstalk‐mediated immune escape. A mouse model of alcohol pretreatment was used. Mice were intragastric with 20% / volume (alc/vol) alcohol daily for 2 weeks, then different colon cancer cell lines (BALB/c mouse CT26 and C57BL/6 mouse MC38) were injected into the spleen to induce metastatic hepatic metastasis. After 10 days, it was found that there was no significant difference in tumor growth in the spleen (primary site) in the alcohol group compared to the control group. However, the alcoholic tissue on the liver metastases showed significant tumor enlargement (Figure 10b). In addition, it was discovered that there was no notable variation in liver IL‐6 within the alcohol group. However, there was an increase in serum IL‐6 levels, indicating that the heightened IL‐6 might originate from an organ outside the liver. The micrometastases of the liver were observed under the microscope 48 h after the injection of MC38 cells into the spleen. In addition to the upregulation of LCN2 in hepatocytes, it also promoted the infiltration of strongly positive LCN2 cells. These cells were identified as neutrophils. Alcohol signature signals IL‐6‐JAK‐STAT3, LCN2, and LY6GC are activated in the liver to promote tumor progression (Figure 10c). Alcohol‐induced hepatocyte LCN2 upregulation and neutrophil infiltration were inhibited by knockout of hepatocyte Stat3 (Figure 10d) or systemic IL‐6 (Figure 10e), suggesting a regulatory relationship. Furthermore, the signaling pathway involving IL‐6‐JAK‐STAT3 is heightened, along with increased expression of LCN2 and LY6G5C mRNA, which are both associated with an unfavorable cancer prognosis (Figure 10f). This study demonstrated that alcohol‐induced hepatocyte STAT3 phosphorylation by up‐regulating hepatocyte IL‐6R and extrahepatic IL‐6. As a result, liver cells upregulate and secrete LCN2, inducing neutrophil recruitment and the transition of cancer cells' stroma to epithelium. The immunosuppressive niche of neutrophil‐guided T cell depletion for cancer cell sedimentation (Figure 10g).^[^ ^203^ ^]^

## 4 Conclusion and Prospect

The world still faces a severe cancer threat, especially in developing countries where the incidence and mortality rates of cancer are still increasing year by year. At present, cancer treatment mainly includes surgery, chemotherapy, radiotherapy, and immunotherapy. Surgery is the preferred treatment for early‐stage cancer, which achieves the treatment goal by removing tumor tissue. However, for advanced cancer or metastatic cancer, the effect of surgery is limited. Chemotherapy kills rapidly dividing cancer cells through drugs. However, chemotherapy drugs can also kill normal dividing cells, such as scalp cells and digestive tract cells, thus causing a series of side effects, such as nausea, vomiting, hair loss, etc. Radiotherapy uses high‐energy radiation to kill cancer cells or prevent their growth. Although radiotherapy is very effective for some types of cancer, it may also cause side effects such as fatigue, skin irritation, and memory problems. Immunotherapy, such as using PD‐1 inhibitors, stimulates the patient's own immune system to attack cancer cells. However, current immunotherapy is not suitable for all types of cancer, and the treatment effect varies from individual to individual. The current situation of cancer treatment exposes the inadequacies of current cancer treatment programs and still needs to develop safe and efficient cancer treatment programs.

With the rapid development of fields such as genomics, immunology, and artificial intelligence, future cancer treatment is expected to achieve more precise and personalized treatment. The development of genomics allows us to more deeply understand the genetic basis of cancer, thereby enabling the development of more targeted treatment strategies. For example, specific drugs or treatment plans can be customized based on the patient's genetic mutations. Immunotherapy is one of the important directions for future cancer treatment. Current research is exploring ways to enhance the anti‐cancer capabilities of the immune system and how to overcome the escape mechanisms used by cancer to evade the immune system. Additionally, combining immunotherapy with other treatment methods, such as chemotherapy and radiotherapy, may result in more powerful synergistic effects. The application of artificial intelligence and machine learning in the medical field will also bring revolutionary changes to cancer treatment. By analyzing large amounts of medical data, artificial intelligence can help doctors diagnose cancer more accurately, predict disease progression, and develop optimal treatment plans for patients. Additionally, artificial intelligence can also be used to develop new drugs and treatment methods, as well as monitor patient outcomes and side effects. The decline of the total incidence rate of cancer in the United States and other developed countries in recent years shows that prevention, early diagnosis, and early treatment are effective in reducing the incidence rate and mortality of cancer. The reminder to humanity is that the new generation of cancer therapies should shift from an era dominated by surgery to an era dominated by prevention and intelligent precision treatment.

In recent years, with the advent of the One Health concept, it has been emphasized that health is a comprehensive concept, and the risk of cancer is closely related to an individual's lifestyle habits. By increasing the awareness of cancer risk among the population and enhancing individual health management, the global incidence of cancer can be greatly reduced. Therefore, we propose the HHM concept, advocating for individuals to develop a healthy lifestyle, enhance the body's immune function, and reduce the incidence of cancer through the management of psychological health, dietary habits, nutritional intake, and exercise habits. Psychology, nutrition, and exercise, as three components of HHM, influence each other and form a complex system. First, moderate exercise can increase energy consumption, reduce the risk of obesity, and promote appetite, thus improving dietary habits. At the same time, a healthy diet can also provide the body with enough nutrition and energy, making the body more energetic to engage in exercise. Second, research indicates that unhealthy dietary habits are associated with psychological issues such as depression. Conversely, good dietary habits can improve mood and alleviate symptoms of anxiety and depression. Lastly, exercise and mental state also affect each other. Regular exercise can stimulate the secretion of dopamine and other neurotransmitters in the brain, thereby improving mood and reducing symptoms of anxiety and depression. In addition, physical activity can enhance self‐confidence and self‐esteem, strengthening stress resilience. The three elements complement each other and collectively enhance bodily functions and anti‐tumor immunity. To maintain a healthy lifestyle, it is necessary to consider these three aspects comprehensively and take appropriate measures to promote a positive cycle among them. The HHM concept is also applicable to cancer patients. By paying attention to the psychological, nutritional, and physical health management of cancer patients, helps them maintain a positive attitude, quickly rebuild immune function, and achieve better prognosis and rehabilitation effects. HHM is a personalized comprehensive prevention and treatment plan that focuses more on individual physical and mental health. Its promotion will increase human awareness of self‐health management and provide new ideas for the prevention and treatment of cancer for the new generation.

## Conflict of Interest

The authors declare no conflict of interest.
